# Interplay of Electronic Orders in Topological Quantum Materials

**DOI:** 10.1021/acsmaterialsau.4c00114

**Published:** 2024-11-25

**Authors:** Christian Stefan Gruber, Mahmoud Abdel-Hafiez

**Affiliations:** † Uppsala University, Department of Physics and Astronomy, Box 516, SE-751 20 Uppsala, Sweden; ‡ Center for Advanced Materials Research, Research Institute of Sciences and Engineering, University of Sharjah, Sharjah 27272, United Arab Emirates; ¶ Department of Applied Physics Astronomy, University of Sharjah, Sharjah 27272, United Arab Emirates; § Physics Department, Faculty of Science, Fayoum University, Fayoum 63514, Egypt

**Keywords:** topology, topological insulator, novel materials, Hall effect, superconductivity, surface effects, semimetal, electronic band structure

## Abstract

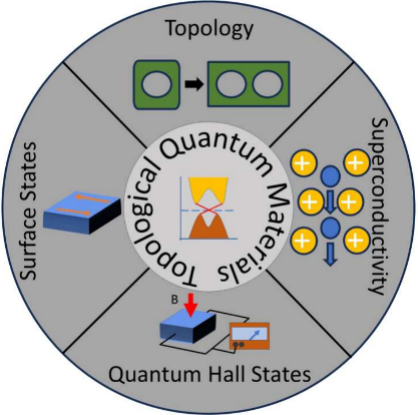

Topological quantum materials hold great promise for future technological applications. Their unique electronic properties, such as protected surface states and exotic quasi-particles, offer opportunities for designing novel electronic and spintronics devices and allow quantum information processing. The origin of the interplay between various electronic orders in topological quantum materials, such as superconductivity and magnetism, remains unclear, particularly whether these electronic orders cooperate, compete, or simply coexist. Since the 2000s, the combination of topology and matter has sparked a tremendous surge of interest among theoreticians and experimentalists alike. Novel theoretical descriptions and predictions as well as complex experimental setups confirming or refuting these theories continuously appear in renowned journals. This review aims to provide conceptual tools to understand the fundamental concepts of this ever-growing field. Superconductivity and its historical development will serve as a second pillar alongside topological materials. While the main focus of this review is topological materials such as topological insulators and semimetals, topological superconductors will be explained phenomenologically.

## Introduction

Topological quantum materials make up a class of materials that exhibit topological properties in their electronic band structures. These materials have attracted significant attention in condensed matter physics due to their unique electronic properties and potential applications in quantum computing and electronics. Some aspects of topological materials (TMs) were already theoretically described as early as the 1970s in 1D and 2D Ising models of superconductors by Thouless and Kosterlitz in what is now known as Kosterlitz–Thouless phase transitions.^1^ Eventually they shared the Physics Nobel Prize in 2016 with Haldane. In contrast to other types of materials, TMs often have an edge over other types of materials with regard to predictability: larger sized TMs have greater robustness against disorganization of the periodic lattice than conventional ones. They can therefore maintain the material’s electronic structure better.^2^ The topic of TMs is a vast and quickly growing field, as it promises novel phenomena, materials, and many applications in industry alike. Novel materials include topological insulators (TIs), magnetic topological insulators (MTIs), topological semimetals (TSs), and chiral crystals (CCs).^3−5^ While many consider the discovery of the quantized Hall effect, later more often referred to as the quantum Hall effect (QHE) in 1980 by Klaus von Klitzing, as a birthdate to topological states of matter, many theorists as well as experimentalists have revolutionized the field over and over again.^6−8^ While some TMs such as TIs have been extensively studied,^9^ fields such as CC especially^10^ (if not all) require further resources. As for additional literature, the reader is also referred to refs (11) and (12).

**Figure 1:**
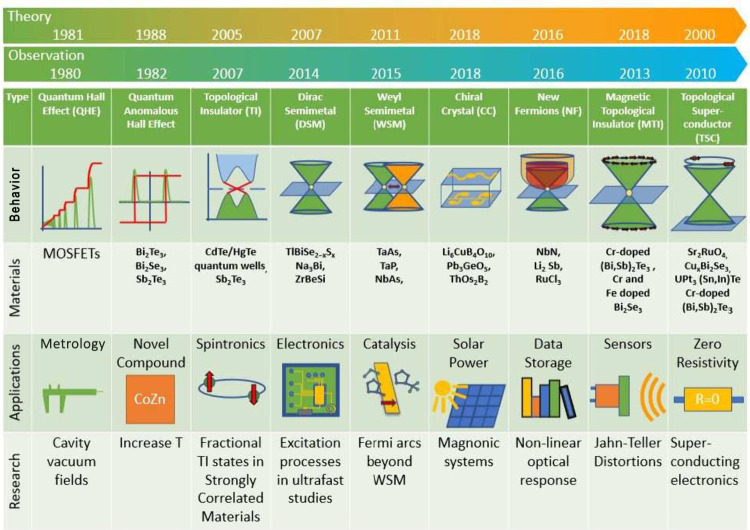
Topological phenomena and materials. In the topmost arrow-bar, the year of the paper of the theoretical prediction is given, followed by the experimental discovery (in topological terms). Additionally, the table gives insight into the currently most interesting characteristic topological phenomena or materials, including diagrams showing characteristic conductance diagrams (only applies to QHE and QAHE) or the energy dispersion relations, qualitatively. Furthermore, exemplary applications and current topics of research are provided. Data taken from refs (3, 8, 10, 17−32).

To show the already extensive family of TMs and phenomena that have emerged over the previous decades, Figure 1 gives an overview of this exciting new subbranch of solid state physics, starting with the QHE in 1980.^6^ The effect had not been predicted theoretically.^13^ The QHE as well as the quantum anomalous Hall effect (QAHE, discovered in 1988) are only given as two examples of the many topological phenomena known today.^14^ Other topological classes include the quantum spin Hall effect (QSHE)^15^ and the fractional quantum Hall effect (FQHE).^16^

The behavior of materials and effects can be seen in the corresponding row “Behavior” in Figure 1. The QHE effect follows characteristic longitudinal and transverse resistivity ρ_*xx*_ and ρ_*xy*_ curves, discussed in more depth in a following section. Additionally, the characteristic diagram of the QAHE is shown as well with ρ_*xy*_ in red and ρ_*xx*_ in green. Diverting the attention from the characteristic behavior of the QHE and QAHE, we then go on to show the characteristic electronic dispersions of different TMs, starting with the oldest of them: the TI with spin–orbit coupling (SOC). Furthermore, the 3D equivalent to the electronic structure of graphene is shown: a Dirac semimetal with its two cone-like dispersions meeting at the Fermi level. From there, when certain symmetries are broken, it is possible to construct a Weyl semimetal. The electronic properties of a chiral crystal are very different to those of the previously described materials, as shown in the schematic binding energy cuts of the electronic spectrum. Novel kinds of electronic structures with spin dependencies are shown for the new fermions, magnetic topological insulators, and topological superconductors, which are all rather recent developments in science.

On the materials side, a multitude of materials such as TIs, semimetals (SMs), CCs, and MTIs as well as specifically topological superconductors (TSCs) will be covered here, if only in a phenomenological and introductory manner. We will later observe that many phenomena are often very similar to each other and hence also to possible applications. Of such character is the relationships between the QAHE with MTIs and QSHE with TIs. Even though Figure 1 suggests a strict categorization, applications may often overlap. TMs have not yet found their rightful place in engineering and electronics. However, to construct smaller, more efficient, and faster devices, it will be hard to dismiss promising topological technologies, an example being conventional field-effect transistors (FETs) being replaced by topological FETs.^33^ Other promising fields of applications include metrology,^34^ novel compounds,^20^ spintronics and electronics,^33,35^ catalysis (reduction, adsorption, etc.),^36^ data storage and conversion,^37^ sensors,^38^ and especially quantum computing applications for TSCs.^39^Figure 1 depicts the chronology of topology by means of categorization: Sr_2_RuO_4_ was already discovered to be superconducting in 1994;^40,41^ however, it was only discovered much later to be of topological nature and named to be “topological” in 2015.^42^

This review is divided into two main sections. First, we will address the fundamental concepts of topology in mathematics, introducing its application to materials science. We will then explore essential topological effects in materials, such as the QHE and the QSHE, acknowledging the wide array of topological effects known today.^15^ Additionally, various TMs will be discussed in a phenomenological context. In the second section, we will delve into the history and development of superconductivity. Finally, we will examine these concepts in the section on TSCs, examining how topology in materials intersects with superconductivity.

## Fundamental Concepts of Topology

Topological phases refer to unique states of matter characterized by the topological properties of their wave functions or energy spectra. These phases emerge in condensed matter physics and significantly impact material behavior. Topology provides a framework to classify whether two wave functions (such as those of electrons or quasi-particles) can be connected adiabatically, meaning through infinitesimally small, continuous changes. If one is able to transform a wave function adiabatically from one to another, these two objects (wave functions) belong to the same topological class (called a genus).^41,8^ As an example, a torus may be squeezed and deformed; however, in order for something to be called a torus, it must have one hole (one hole specifically) at all times (to remain in the same class of objects). Otherwise, the classification of a torus no longer holds true.

When examining possible new TMs, one is thus looking for invariant/breaking symmetries that best describe the macroscopic behavior. Nontopological quantum materials typically have band structures that can be described by conventional band theory. In these materials, the energy bands and electronic states within them can be continuously deformed without changing the topological properties. In contrast, topological quantum materials possess band structures with nontrivial topology, characterized by the presence of protected surface or edge states, nontrivial band crossings, or nontrivial topological invariants, such as the Chern number *C* or *Z*_2_.^43,3^

The Chern number is a prominent example of classifying a topological invariant. It does not change under an adiabatic evolution. It may be computed by taking the integral of the Berry curvature over the Brillouin zone, where Ω_*xy*_ is the Berry curvature.
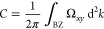
1The Berry curvature originates from a rotation (curl) of the Berry phase, which is a phase being picked up by a state under cyclic evolution. A quantum mechanical state that changes slowly enough (adiabatically) under a time-dependent Hamiltonian does not undergo sudden energy-eigenstate transitions but rather adjusts to the changing Hamiltonian. Additionally, the phase *e*^*iϕ*(*t*)^ that is being picked up will not show in the expectation value of the Hamiltonian. Rather, the Berry phase becomes relevant upon superposition phenomena of two states.^44^ That is also why there is an ambiguity in the overall phase. One may choose it freely; however, its relative phase to other states’ phases is crucial (superposition). In that regard, the Berry phase is very similar to the vector potential, as known in classical electrodynamics.^45^ The Berry curvature in solids was recently experimentally detected by Schüler et al. by photoemission spectroscopy.^46^

In terms of TMs, if one wants to transition from one topological phase to a topologically distinct one, then the energy gap in the energy dispersion relation must close at some point in the process. If this were not the case, the two phases could be adiabatically connected and thus be the same topological object.^45^ Therefore, if a topological material (e.g., *C*(Cu_*x*_Bi_2_Se_3_) = 1) is positioned on topologically trivial matter (e.g., air, *C* = 0), the energy gap must close, which results in metallic surface states.^43^ The Berry curvature may also be considered as entanglement between the conduction and valence band of the system.^47^

### Symmetries: Parity and Time Reversal

Symmetries, particularly parity symmetry and time-reversal symmetry (TRS), are fundamental to understanding and characterizing topological quantum materials. These symmetries provide a basis for distinguishing different topological phases and predicting their properties. Before delving deeper into the topic, it is essential to explore these two key physical symmetries, as they hold special relevance in topology.

In condensed matter physics, physical systems are often classified based on the symmetries they preserve or break. For instance, topological insulators and superconductors exhibit unique properties, depending on their symmetry classifications. The interplay between parity and time-reversal symmetries can lead to novel phenomena such as the protection of edge states in topological insulators or the emergence of Majorana fermions in topological superconductors. Understanding these symmetries and their implications helps in the comprehensive characterization of topological quantum materials, guiding both theoretical predictions and experimental discoveries. As an example, crystals break translational symmetry.^48^ Thus, two symmetries will be discussed: parity and time reversal.

The parity operator acting on a wave function is often seen as a mirror in space, where *x* → −*x*, *y* → −*y*, and *z* → −*z*.^49^ If a quantum mechanical system (such as the electronic structure) remains unchanged under a parity transformation, the system is considered to be “even” parity. If, under a parity transformation, the system reverses its sign (negative to positive or vice versa), it is considered “odd” parity. The total angular momentum *J* for a spinless free particle would be such a candidate for even parity, whereas a free fermion has odd parity.^50^ The fermionic parity operator tells us if we have an odd or even number of fermions we are working with.^51^

Time-reversal symmetry is a fundamental symmetry that relates the forward and backward evolutions of a physical system. It states that if a system evolves from an initial state to a final state, the time-reversed evolution should also be allowed. In the context of topological quantum materials, time-reversal symmetry plays a central role in protecting and characterizing various topological phases.^49^ Speaking in terms of classical mechanics, entropy would be TRS breaking, except for when in equilibrium. In quantum mechanics, spins flip under a time-reversal operator, thus often (though not always, as we will see) leading to TRS breaking. The cause of this spin flip may be made in a semiclassical approach: any magnetic moment that flips under a time-reversal operator thus also spins.^52^ The QHE is a good example for TRS breaking, while the QSHE is prominent for time-reversal invariance (TRI). Beyond the phenomenological approach, the time-reversal operator applied upon a wave function results in the complex conjugate of it.^7^

Even though symmetry breaking schemes at first might sound rather nonintuitive and merely theoretical, Bernevig, Hughes, and Zhang were able to demonstrate the time-reversal invariance in HgTe/CdTe quantum wells as early as 2006, and the quantum Hall effect (despite not knowing the fundamental topological mechanisms then) has been around since the 1980s.^6,53,54^

### Majorana Zero Modes

Majorana zero modes (MZMs) are quasi-particles that have attracted significant attention in the field of topological quantum materials, particularly in the context of topological superconductors. A Majorana zero mode refers to a quasi-particle excitation that is its own antiparticle. MZMs are fermionic modes that have zero energy (hence, “zero mode”) and exhibit nontrivial braiding statistics. Unlike conventional fermions, which can be either occupied or unoccupied, MZMs are regarded as a superposition of electrons and holes.^25,55^ Neutral excitations in the bulk superconductor may then be identified as Majorana fermions. It is here where many TMs exhibit interesting (surface) phenomena.^25^

Several experimental techniques have been employed to detect and probe MZMs in topological quantum materials. One common approach is tunneling spectroscopy and scanning tunneling microscopy (STM),^56^ which can reveal the presence of localized zero-bias conductance peaks associated with MZMs. Other techniques, such as Josephson junctions and interferometry, have also been utilized to study MZMs and their properties.^57,58^

## Hall Effects

Equipped with a foundational understanding of topology, we can now delve into the fascinating realm of various Hall-related effects. These phenomena bridge the theoretical framework of topology with experimental materials, providing concrete examples of the topological principles in action.

The journey begins with the classical Hall effect (HE), discovered by Edwin Hall in 1879. The Hall effect describes the deflection of electrons under the influence of an electric field due to the Lorentz force when a perpendicular magnetic field is applied, resulting in the generation of a Hall voltage.^59^ This discovery laid the groundwork for an entire field of study, becoming a fundamental concept in condensed matter physics.

Since then, the Hall effect has evolved into a cornerstone of modern research, leading to the discovery of several related phenomena that showcase the significance of topology in real materials. The quantum Hall effect (QHE) demonstrates quantized Hall conductance in two-dimensional electron systems subjected to low temperatures and strong magnetic fields. The quantum anomalous Hall effect (QAHE) occurs in certain magnetic materials without an external magnetic field, driven by intrinsic magnetic order and strong spin–orbit coupling. The quantum spin Hall effect (QSHE) involves the generation of spin-polarized edge states in topological insulators where spin currents flow without dissipation. Lastly, the fractional quantum Hall effect (FQHE) reveals the presence of quasi-particles with fractional charge and statistics in two-dimensional electron systems under extreme conditions.

These Hall-related effects not only highlight the quantized nature of electronic transport but also underscore the profound impact of topology on condensed matter physics. By examining these phenomena, we gain insight into how topological properties manifest in experimental systems, paving the way for future advancements in the study and application of topological quantum materials.

### Quantum Hall Effect (QHE)

A century after the discovery of the fundamental HE, 1982 brought a breakthrough in condensed matter physics by Klaus von Klitzing with the discovery of the QHE, which fundamentally shifted the understanding of the HE. It was discovered that at sufficiently low temperatures and high magnetic fields, the Hall resistivity of a material is quantized. Thus, a first topological invariant necessary to describe Hall resistivity in the QHE had been discovered.^54^ His findings also marked the beginning of the TMs. He was awarded the Physics Nobel Prize in 1985.^6^

The resistivity of the quantum Hall effect may be computed as follows, where *R*_Hall_, *h*, *C*, and *e* are the Hall resistance, the Planck constant, an integer, and the electron charge, respectively:
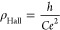
2From this equation alone, it becomes clear that the resistivity (and thus conductivity) is quantized by an integer number *C*, the Chern number.

**Figure 2:**
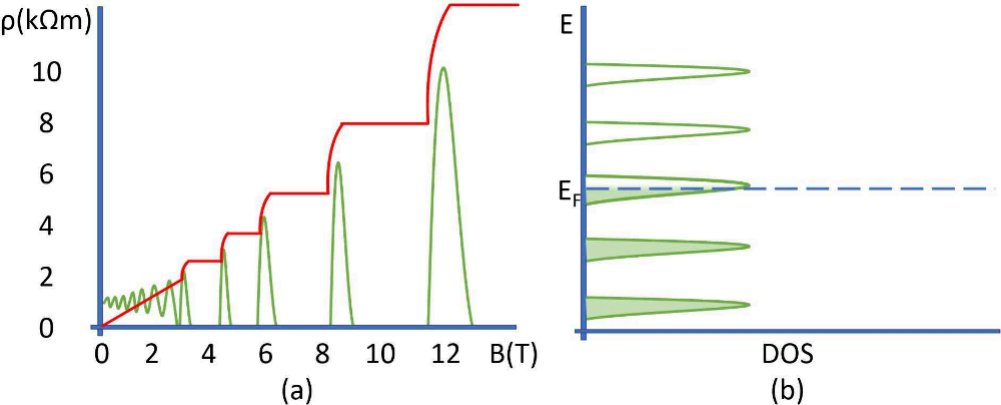
Qualitative characteristic functions of the QHE dependent on magnetic field strength *B*. (a) Schematic characteristic functions of the transverse (red) and longitudinal (green) resistivity. (b) Quantized Landau levels under a high magnetic field *B*. Adapted from ref (30). Copyright 2019, Purdue University.

To get a better feeling of the effect and how the Chern number may be determined through experiments, we look at Figure 2. The two subfigures explain the QHE by the characteristic functions (red and green) and show the typical density of states (DOS) at high magnetic fields derived by Landau.

Before we investigate what happens in the schematic characteristic function of the QHE, we must first understand Landau levels (Figure 2b). Quantized Landau levels arise due to large magnetic fields (magnitude of T) that squeeze the DOS and may be derived by the Hamiltonian of a 2D film with a perpendicular magnetic field acting upon it.^60^

The separation between the levels is proportional to the cyclotron frequency ω_c_, where *q*, *m*, and *B* correspond to the charge and mass of the electron and the applied magnetic field, respectively:
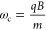
3One realizes instantly that the distance between the Landau levels shrinks with decreasing *B*. Additionally, Landau levels broaden due to electron–electron interaction and impurities of the system.^61^ When the Fermi energy (denoted as *E*_F_) crosses a Landau level, both longitudinal (green in Figure 2a) and transverse (red) resistivities decrease instantaneously. The broadened Landau level acts as both the conduction and valence band, thus allowing current to flow and resistivity to drop. By further decreasing *B*, this Landau level shifts further down until it is no longer at the Fermi level, resulting in fully insulating materials. At low *B*, Landau levels become indistinguishable from each other due to their proximity and are positioned beneath the Fermi level. The longitudinal resistivity occurs in true quantization, while the transverse resistivity occurs in plateaus, due to impurities of the system.^6^

A more detailed explanation of the QHE and Landau levels may be found in refs (6, 13,) and (62). Today, the QHE is applied in fields such as metrology.^34^

### Quantum Anomalous Hall Effect (QAHE)

One might pose the question of whether the QHE can also be induced without strong outer magnetic fields. Such behavior may indeed be induced by an appreciable crystal structure and chemical composition. Such an effect is known as the quantum anomalous Hall effect (QAHE).

Building upon the concept of the QHE, but by no means as famous, the QAHE is the quantized equivalent to the anomalous Hall effect (AHE) and closely related to MTIs. In the AHE, the deflection of electrons in a material may occur, even if no outer (or inner) magnetic field is present. The underlying reason for this nonquantized anomaly is ferromagnetism, SOC, and disorders, as proposed by Yu et al.^17,63^

Like the QHE, the QAHE arises from a nonzero Chern number. The nonzero Chern number (a result from TRS breaking) may, however, not stem from a magnetic field, which would again lead to Landau levels. As shown in eq 1, to obtain the Chern number, one integrates over the Brillouin zone and its occupied bands. Due to other contributions, metals, however, will not exhibit integer Chern numbers and have trivial topology. Few materials fulfill all requirements (also called Chern insulators);^63^ however, some material families exist: magnetically doped topological insulators like (Bi,Sb)_2_Te_3_, thin films of the magnetic topological insulator MnBi_2_Te_4_, topological semimetals,^5^ and Moiré materials formed from graphene and transition metal dichalcogenides.^65^

### Quantum Spin Hall Effect (QSHE)

The QHE requires a magnetic field to induce a quantum Hall state (QHS). While the implications of such a system are remarkable, the applied magnetic field breaks the TRS of the system. Soon after its discovery, scientists started working on a spin-channel separated equivalent of QHE, which would allow TRS to remain intact. However, it remained unclear whether one could realistically separate the spin current channels and tackle possible impurities.^39^

In 2005, however, equipped with a new topological invariant *Z*_2_ (comparable to the Chern number but suited for TRI systems), Kane and Mele predicted that the QSHE in graphene would keep TRI but also remain in a QHS.^3,66^ They took the crucial step of splitting up the Hamiltonian of the QHS into two separate ones. This resulted in two identical copies of the Hamiltonian but with opposite spins (up and down). Thus, they took into account the spin orientation of the electrons. The overall system remains in its original QHS because upon the time-reversal operation, the two Hamiltonians flip their spin orientations but result in each other.^3^

In two dimensions, there exist two resulting edge states, one for each spin channel. No net current will flow if an electric field is applied since the electrons of different spin channels move in opposite directions. Net spin currents, however, do flow.^3^ These theoretical predictions were soon confirmed experimentally in 2006 by Bernevig et al. in HgTe.^53^

### Fractional Quantum Hall Effect (FQHE)

Yet another contribution to the canon of Hall-related effects was made by Laughlin, Störmer, and Tsui. They received the Nobel Prize in 1998 for their work on a fractional quantization of the Hall conductance.^67^ They discovered that the integer plateaus, as shown in Figure 2, may also take fractional values in 2D electron gases that condensate into a 2D quantum fluid.^68^ The FQHE primarily arises in strongly correlated systems.^63,69^ Consequently, eq 2 must allow for noninteger charges. In a nutshell, this may be explained by a quasi-particle (instead of electronic) excitation, making it necessary to introduce fractional (going beyond Bose and Fermi) statistics to the problem.^70^ An example material (due to its high mobility) is GaAs and graphene.^71^ Generally, it may also be observed in Weyl semimetals (WSMs).^72^

## Topological Materials

Now that the understanding of Hall-related effects is well established, TMs themselves will be the main focus of this section. It will start with perhaps the most famous of the TMs: TIs. Then, topological semimetals (TSMs) shall be studied a bit more in depth, focusing on Dirac semimetals (DSMs) and WSMs. Finally, CCs and MTIs will be tackled.

### Topological Insulators (TIs)

**Figure 3:**
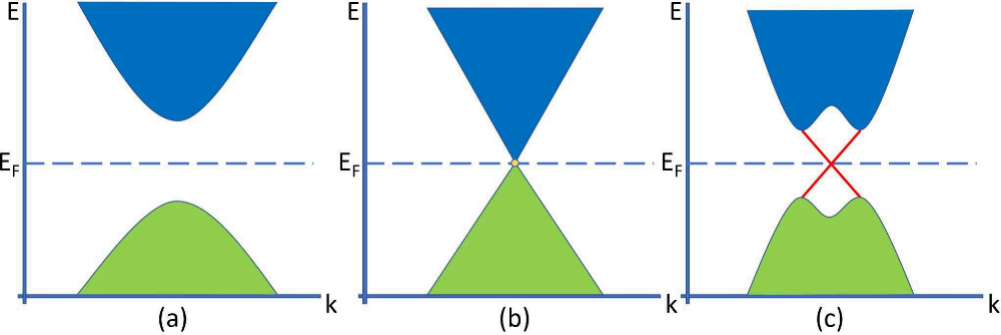
Schematic band structures (green = valence band, blue = conduction band, and red = surface states) of (a) a regular insulator, (b) a Dirac cone with a Dirac point (in yellow), and (c) a TI. The TI exhibits surface states, indicated in red, as well as band inversion due to strong SOC. Adapted with permission from ref (64). Copyright 2021, Springer Nature.

To better understand the practical consequences of the Chern number and of topology in general, we shall investigate an arbitrary topological insulator: in a regular insulator, the band gap between the valence and conduction bands is too large; hence, no electrons are excited into the conduction band (readily described by classical band theory). However, a topological insulator also displays interesting phenomena on the surface. These so-called surface states/bands can exhibit conducting, superconducting, magnetic, antiferromagnetic behavior, etc. That is what makes topological quantum materials different from trivial materials.^3^ These surface bands may often be characterized by Dirac cones, which may become clearer with Figure 3. These surface bands in the energy dispersion relation closely link to symmetry protected states as discussed with topology.

As can be seen in Figure 3a, the trivial band structure of an arbitrary insulator is shown. Figure 3c displays the typical band structure of a topological insulator. Using classical band theory, the valence band and conduction band should readily overlap, resulting in a conductor. However, due to so-called band inversion, a very special band structure arises. Band inversion stems from the spin–orbit coupling (SOC) of the system. SOC describes the magnetic field induced by the moving atomic nucleus acting on the electron (and its spin state) in the reference frame of the electron.^73^ While the band gap between the two bulk phases of the insulator is still too wide for any electrons to transition between the valence and conduction band, conducting surface states occur (red). The first experimental discovery of such surface states was made in 2008 by Hasan et al.^74^ As already mentioned, the underlying theoretical concepts were developed by Kane and Mele in terms of the quantum spin Hall effect.^3^

Figure 3b depicts a DSM. One may see the DSM as an intermediate material between a trivial insulator and a topological insulator. DSMs have gapless energy bands and are topologically as well as symmetrically protected.^19^ DSMs will be tackled in the next section.

Over the previous years, yet another subdivision of TMs research has emerged: topological Kondo insulators (TKIs).^75^ This subfield brought together two fields of physics: heavy fermion and topological systems. The Kondo effect itself was already discovered in the 1930s, describing the electron–electron interaction, which is stronger than the kinetic energy of the electrons in heavy fermion systems. That in turn gives rise to high electron–electron correlations and exotic phenomena. There exists a characteristic Kondo temperature *T*_K_ (not to be mistaken by the critical temperature *T*_C_ of SCs) above which the material behaves like a metal with localized magnetic moments (also referred to as magnetic impurities in the lattice) because electrons only scatter weakly off those impurities at high temperatures. Below *T*_K_, scattering becomes a dominant phenomenon, leading to the creation of spin singlets between the magnetic disorders and electrons. That in turn leads to distinctive band gaps in the energy dispersion relation.^75^ The first TKI to be discovered was SmB_6_ in the late 1960s.^4,75^

### Topological Semimetals (TSMs)

Another interesting exotic topological phenomenon is TSMs. Among the most famous are DSMs and WSMs.^5^ In theory, a distinction between the two may be considered almost trivial: electrons in DSMs follow the Dirac equation,^76^ while electrons in WSMs follow the Weyl equation.^77^ Phenomenologically, however, they differ substantially. Both Weyl and Dirac fermions assume vanishing effective masses of the fermion (in our case, the electrons in the crystal).^76,77^ As opposed to TIs, topological semimetals already have bulk conductivity but may exhibit similar or identical metallic topological surface states.^5^

#### Dirac Semimetals (DSMs)

**Figure 4:**
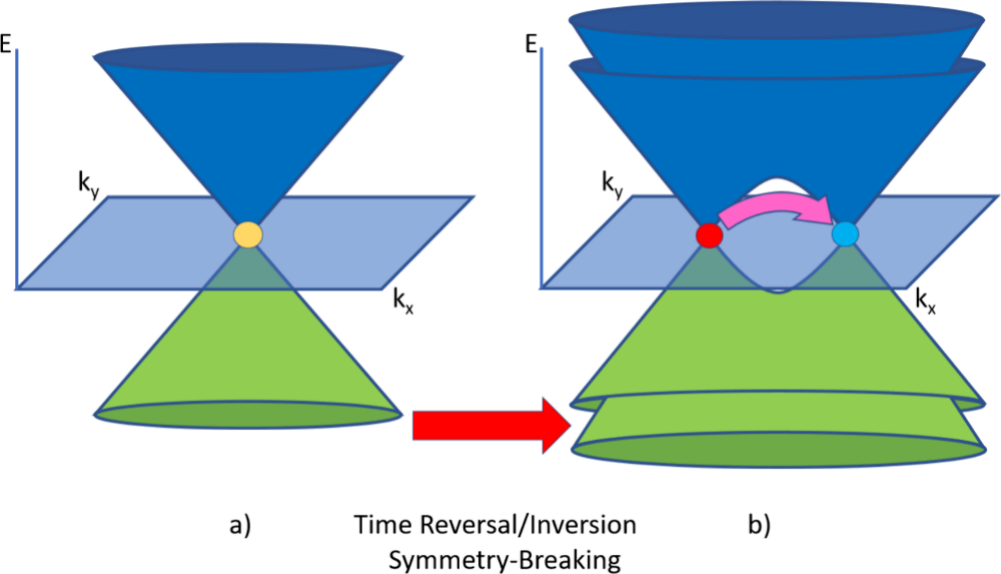
Three-dimensional schematic band structures of (a) DSM and (b) WSM. In DSM, the conduction (blue) and valence (green) bands are connected at a Dirac point (yellow). When TRS or inversion symmetry is broken, the Dirac point splits into two Weyl points of opposite chirality, which are connected at the surface through a Fermi arc (pink). Modified from ref (31). Copyright 2018, Technische Universität Dresden.

A DSM-like band structure is composed of two Dirac cones, one of them being inverted and connected by a so-called Dirac point, which is ideally placed as close as possible to the Fermi level, as shown in Figure 4.

The energy dispersion in the band structure thus exhibits 3D linear (conical) behavior very much like the 3D equivalent to graphene.^47,78^ However, graphene does not have any topologically protected surface states. If put on a substrate, graphene’s energy gap opens up. Due to topological protection, that would not happen to a DSM.

Like in TI, spin and momentum are also locked up in topological semimetals. Oftentimes (such as in Cd_3_As_2_), the conical band structure arises due to the strong dependence of the effective mass of the system on the electron concentration and SOC.^5^

While research in the example of Cd_3_As_2_ had already started in the 1930s as the interest of scientists shifted to semiconductors, it was only in 2013 that Wang et al.^79^ predicted Dirac cones in materials.^5^ Previously, other models like the Kane model on semiconductors described the material quite well via *k* · *p* perturbation theory.^80^ If the band gap is small compared to the energy scale of the material, conical structures like those in DSM in the band structure arise. However, these models did not incorporate the essential protected surface states. Wang’s prediction was already verified a year later by Rosenman et al.^81^

#### Weyl Semimetals (WSMs)

WSM has received favorable attention in previous years, as quantum computing and spin transport applications are to be expected in the future. WSM superconductors already exist.^47^ Similarly to TI, band inversion occurs in WSM, as may be seen in Figure 4, when TRS or inversion symmetry is broken in a DSM.

When either of those symmetries are broken, the Dirac point ceases to exist/is split up, and from that point on, two doubly degenerate Weyl points^82^ of opposite chirality (positive or negative, such as in dipoles) emerge. The Berry curvature at these points is either positive (source) or negative (sink) in chirality. This makes an uneven number of Weyl points impossible since otherwise the Berry curvature would be confronted with divergence issues.^47^ There exists, however, one exception: when TRS and inversion symmetry endure, the Weyl points become degenerate in a single point, once again becoming a Dirac point.^47^ Thus, by tuning the symmetries, one may transition from one topological semimetal to the other. Experimentally, that is not so easily done.^47,79^ Finally, it is usually necessary to bring the Fermi level as close as possible to the Weyl points. That may be achieved via electron doping.^83^

In short, Weyl points, closely connected to the gapless surface states at the Fermi level, are protected by surface topology and are of opposite chirality. Opposite chirality Weyl points are connected by Fermi arcs, which are metallic surface states.^84^

Current research has recently taken a special interest in type-II WSM. This advancement of the original WSM introduces a tilt of the Weyl cone with respect to the Fermi plane and induces the QAHE. The QAHE is caused by a nonzero Chern number that in turn results from the Berry phase that is picked up in the *k*_*x*_*k*_*y*_-plane. Finally, it is noteworthy to mention that interesting phenomena like negative magnetoresistance under parallel magnetic and electric fields and exotic transport properties arise due to the chiral anomaly in WSM.^84,85^

### Chiral Crystals (CCs)

Materials that do not exhibit any rotational or inversion symmetries (including a combination of both, called roto-inversion) may often be classified as CCs, which is in contrast to other materials that have been introduced here. Such a lack of symmetry often leads to a topological band structure.^10^

CCs are interesting because of their exotic optical and magnetic responses. Chiral magnets, for example, could be used to induce skyrmions.^10,86^ A material of the CC family is said to possess a certain handedness with regard to its crystal structure.^10^ Such handedness arises from the massless Weyl fermion (the massless electron following the Weyl equation).^77^

**Figure 5:**
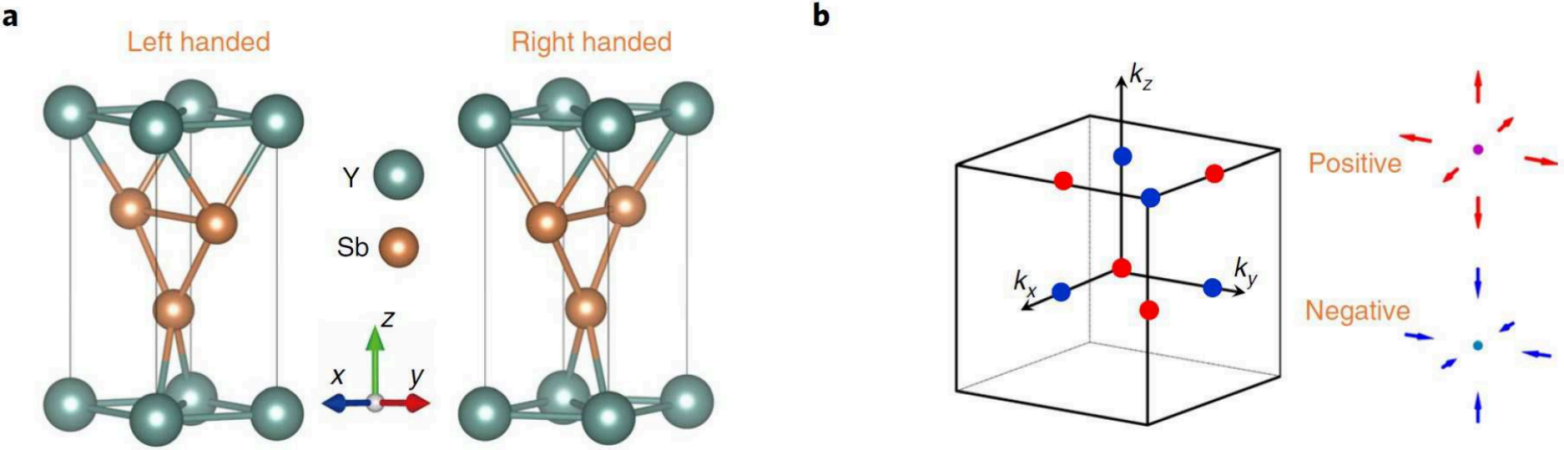
Chiral crystals. (a) Example of inversion and rotation symmetry breaking leading to right and left handed materials. (b) Chiral fermions responsible for topologically nontrivial Chern numbers. Reprinted with permission from ref (10). Copyright 2018, Springer Nature.

The fermion shows certain spin behavior (up or down), which cannot be mirrored, rotated, or inverted. Thus, the spin up (or down) of the electron results in the handedness. This is shown in Figure 5a, where the given crystal structure cannot be mirrored, rotated, or inverted.

To understand CCs, a crucial concept is points of time-reversal invariant momenta (TRIM). Such TRIM points may be considered nodal fermions that sit at specific spots in the Brillouin zone and are the driving effect for various phenomena in CCs. Chang et al.^10^ showed that nodes with nontrivial Chern numbers arise from Kramers degeneracies at TRIM. Kramers degeneracies occur in systems of half-integer total spin. Kramers’ theorem states that there is minimally one other eigenstate of the same energy upon a time-reversal operation of the Hamiltonian if the system’s total spin is a half-integer value.^87^ They found chiral fermions (electrons) of opposite chirality in orthorhombic crystal symmetries, as shown in Figure 5b.^10^

Combining CCs with the concept of semimetals, yielding chiral semimetals such as PdGa, promises yet another interesting path, which may be taken in terms of TMs.^88^

### Magnetic Topological Insulators (MTIs)

Essentially, MTIs have to fulfill some fundamental criteria: ferromagnetic ordering and SOC must be strong enough for a nonzero Chern number to arise (to be topologically nontrivial). TRS breaking is also required, which is achieved by the insertion of (anti-)ferromagnetism into the TI. By such an introduction, gaps open up due to magnetic exchange interactions.^23^ Naturally, MTIs exhibit QAHE behavior^90^ and homogeneous magnetic ordering on the surface.^23^

The history of the QAHE is closely related to the discoveries connected to the class of MTIs.^91^ Initially, researchers were unsuccessful at finding suitable materials exhibiting the QAHE since oftentimes extreme conditions were necessary. Another reason was that bulk bands often mixed with the topological surface states, making numerous materials subsequently unfitting. By doping (Bi,Sb)_2_Te_3_ with Cr, Chang and associates were the first ones to detect the QAHE within a real material. Unfortunately, large magnetic fields as well as temperatures beneath 0.1 K were necessary.^92^ With an increasing quality of synthetization over time, temperatures of up to 2 K became possible. In addition to doping, stacking TI and ferromagnetic heterostructures or introducing magnetic layers in TIs may also lead to MTIs.^91^

## Superconductivity

Since Heike Kamerlingh Onnes’ ability to liquefy helium in 1908 and his subsequent discovery of superconductivity some three years later,^93^ great advancements in the field of superconductivity have been made, and new research fields such as topological superconductors have emerged. This section will investigate the early historical developments of superconductivity and will elaborate on primary milestones of this ever-growing subdiscipline of condensed matter physics and the relatively recent theoretical approaches and experiments conducted specifically in the field of TSCs.

### Early Experimental Findings

After the Dutchman Heike Kamerlingh Onnes had accomplished sufficient cooling of liquefied helium (about 4.14 K) in 1908, he was then interested in the resistivity behavior of pure metals near absolute zero temperature, introduced by William Thomson (Lord Kelvin). According to Kelvin, the resistivity of any pure metal should linearly decline with temperature but peak toward infinity at absolute zero. Kamerlingh Onnes first also supported that theory. Previous cooling techniques had, however, only allowed temperatures as low as 14 K, such that with Kamerlingh Onnes’ new cooling technique, further experimental progress was possible. He soon found that Kelvin’s theory only holds for pure metals such as gold, platinum, and mercury above certain material-dependent threshold temperatures. Resistivity plummets below such temperatures and becomes almost unaffected by a change in temperature by going even lower.^93^ He thus observed the phenomenon of superconductivity for the first time in human history.

Kamerlingh Onnes received the Nobel Prize in Physics in 1913^94^ promptly after his discovery in 1911. Kamerlingh Onnes also reported that relatively small magnetic fields would break the superconducting state.^95^ His discovery would spur solid state physicists up until today since the promise of his discovery was quite clear: loss-free electron transfer occurs across large distances.

**Figure 6:**
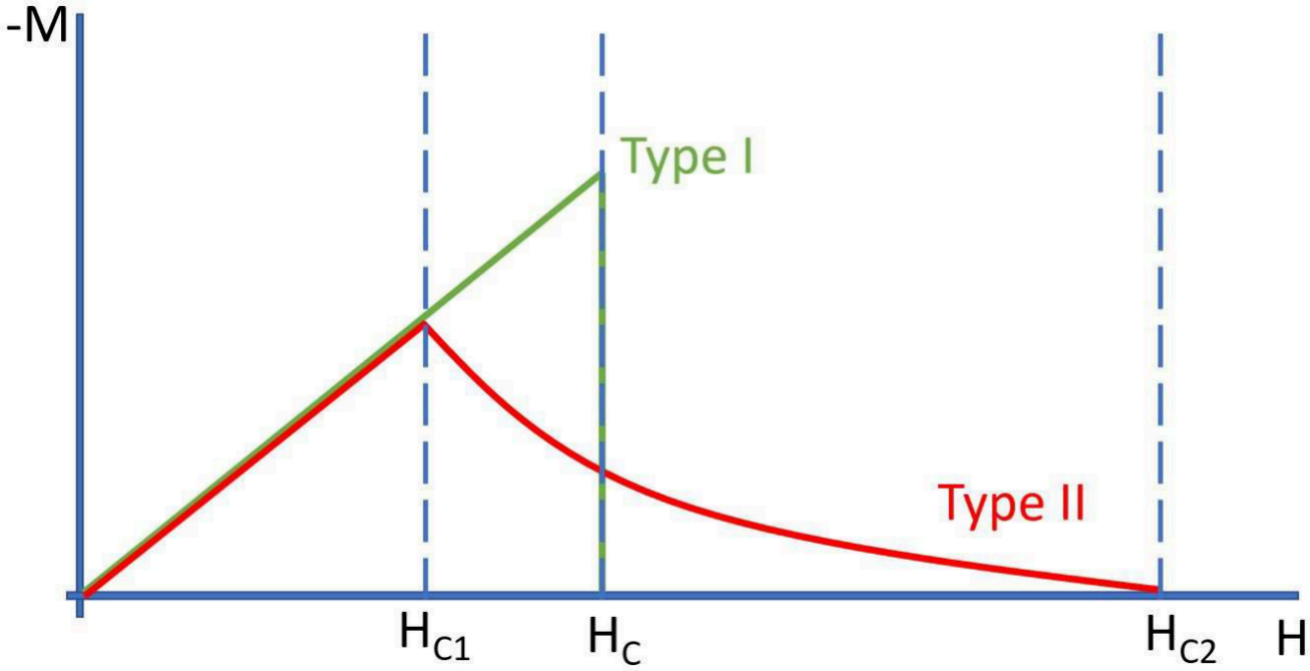
Schematic magnetization curves of type-I and type-II SCs. Adapted with permission from ref (89). Copyright 2019, Elsevier.

Relatively few advancements in the field were made in the following two decades, even though superconductivity in alloys of two nonsuperconducting materials was discovered by de Haas and associates in 1928.^96^ Yet another contribution to the growing field of superconductivity research was the experimental discovery of type-II SCs by Shubnikov et al. in 1937.^97^ This new type of SC had a very interesting property: while type-I SCs’ (all previously discovered SCs) threshold temperature decreased in an increasing magnetic field, type-II SCs exposed a peculiar behavior. While these new superconducting materials behave like type-I SCs beneath a certain magnetic field strength, their superconducting state does not collapse above such a magnetic field. Rather, the magnetization decreases exponentially, as can be seen in Figure 6.

### Meissner–Ochsenfeld Effect

The next great breakthrough came some years later: the Meissner–Ochsenfeld effect. Walther Meissner and Robert Ochsenfeld found that SCs will only allow a given magnetic field acting upon it to enter to a certain depth^98^ (later to be known as the London penetration depth). Meissner and Ochsenfeld found that this maximal penetration depth allows magnets to float above (and beneath) SCs.^98^

While one may look at Meissner and Ochsenfeld’s discovery simply as one in a chain of physics research, one should not underestimate the importance of their findings. For that reason, we focus on the difference between an SC and a perfect conductor.

As the name suggests, the perfect conductor becomes perfectly conducting as the temperature falls beneath its critical value (*T*_C_). However, the perfect conductor still follows Faraday’s law:
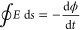
4where *E* denotes the electric field, ϕ is magnetic flux, and *s* is the integral path. The law states that a changing magnetic field through a surface ϕ with respect to time  is equal to the closed path integral of the surface boundary of the electric field *E*. Thus, when the magnetic flux changes, a voltage may be measured along the surface boundary (for example, in a wire).

**Figure 7:**
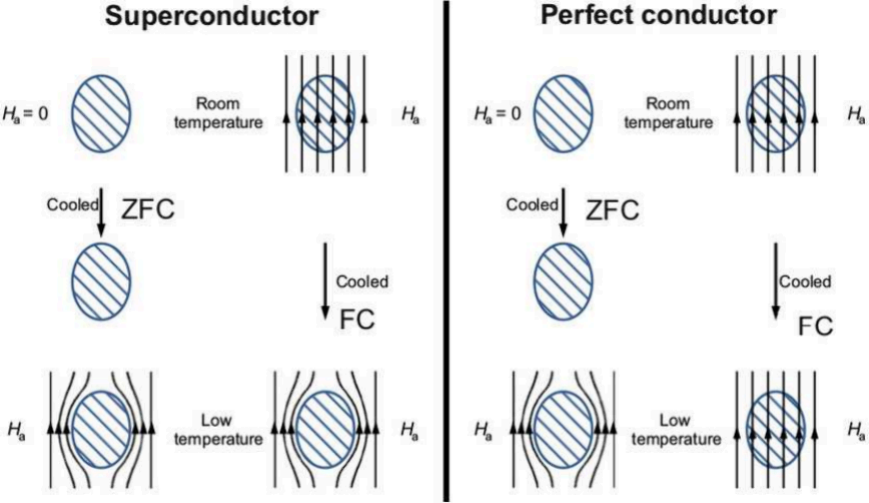
Schematic comparison of a perfect conductor and SC. Reprinted with permission from ref (99). Copyright 2015, Elsevier.

To get a better understanding of the particularity of SCs and the Meissner–Ochsenfeld effect specifically, we shall now study the difference between a perfect conductor and an SC in more depth. We begin with an SC and a perfect conductor at room temperature with and without an applied magnetic field *B* (see Figure 7).

The perfect conductor behaves classically. To explain the perfect conductor, one should start with Maxwell’s equations and that the response to any external electric field in the perfect conductor is instant (requires no time). Thus, given an external electric field, an internal electric field arises simultaneously with the external electric field, hence canceling out the overall electrical field in the conductor. That yields , where *D* = ϵ_0_*E* is the macroscopic polarization (for a linear response). Using Maxwell’s equations, one may compute , and we already know . From these two equations, it becomes clear that the magnetic field must be constant inside the perfect conductor. Therefore, one speaks of a “frozen-in” magnetic field since *B* remains constant and depends on the history of the perfect conductor.^99,100^

The situation is rather different in an SC. The SC’s magnetization does not depend on its previous history. If we apply a magnetic field only after cooling the SC below *T*_C_, the SC behaves as expected. However, if an SC is exposed to an electric field at room temperature and cooled beneath *T*_C_, the SC still expels the external magnetic field: the Meissner–Ochsenfeld effect.^100^

### The Theory behind Experiments

Over the past century, many theorists failed to explain the concept of superconductivity at a microscopic level. Great physicists like Einstein, Bohr, Kronig, and Bloch failed to the extent that their predictions would fit the experiments.^101^ Eventually, eye-opening achievements were made. Upon finding out about Meissner and Ochsenfeld’s discovery, the London brothers noticed the important relation between the electrons in the material and the applied magnetic field. Shortly after, Ginzburg and Landau introduced their famous phenomenological description of superconductivity. Bardeen, Cooper, and Schrieffer (BCS) then succeeded in deriving a more general microscopic description of superconductivity in 1957.^102^

#### London Equations

Spurred by the discovery of the effect discovered by Meissner and Ochsenfeld, Fritz and Heinz London were pioneers in tackling superconductivity in a theoretical manner. Their starting point is classical electrodynamics. They started off with the free electron model being acted upon by the Lorentz force:
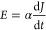
5in which *J*, *t*, and *E* are the current, time, and electric field, respectively. , where *m* denotes mass, *n* is the charge density, and *e* is the electron charge. (However, there is a catch in the acceleration equation, as F. and H. London called it.) As London and London admitted right from the beginning, eq 5 does not account for any friction of the charges at all.^103^

Combining eq 5 with Faraday’s law  and since , one arrives at the following equation:^103^
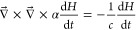
6Using  and integrating with respect to time yields
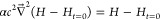
7They noted that the solutions of this inhomogeneous differential equation behave ordinarily inside the SC, whereas one may compute that the solutions exponentially decrease as one moves further from the SC’s surface into the external magnetic field *B*. They further argue that eq 7 gives a too general solution to the problem of the theoretical description of superconductivity. Therefore, they replace  with , resulting in the first London equation.^103^
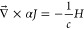
8By solely working with eq 8, they rid themselves of working with a very general differential equation and were able to simplify the abundant set of general solutions to natural behavior, such as the Meissner effect.^103^

London and co-workers further derived the second London equation in a similar fashion. Here, they used eqs 5 and 8. However, they found a component  which is proportional to the time-like component of the four-vector of *j*_*i*_, the relativistic current density. Thus, they gave their differential equation an interpretation by the principle of covariance, an interpretation that observers in all reference frames may correlate the same physical quantities (introduced by Einstein).^104^ With this equation, they postulated the replacement of Ohm’s law (*J* = *σE*, σ being the conductivity):^103^
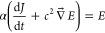
9Finally, they also described a crucial physical property in SCs: the London penetration depth (λ). Combining Ampere’s law with eq 8, one arrives at
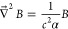
10The solution to this differential equation is obvious. Expressing  in SI units yields the London penetration depth, μ_0_ being vacuum permeability:
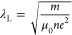
11Even though they described some phenomena of this early stage of theoretical SC investigations quite well, they too were aware of the missing links of their formalism.^105^ They concluded that the electrons shield off the external magnetic field.^95^

#### Ginzburg–Landau Theory

Soon thereafter, further improvements were made by Vitaly Ginzburg and Lev Landau resulting in the Ginzburg–Landau theory in the 1950s. Their theory is based on the free energy of the superfluidity of electrons.^106^ Superfluidity is a quantum mechanical state in which particles lose all their friction.^107^ Using Landau’s theory on second order phase transitions, they described superconductivity by means of a complex order parameter ψ of a second order phase transition related to the free energy of the system. This order parameter made it possible to determine to what extent the material is in the superconducting phase.^108^ It is important to note that any solutions are only nonzero solely in the superconducting phase (below a threshold temperature, denoted as the critical temperature *T*_C_).^109^

First they expanded the free energy *F* of the system in terms of a complex order parameter:
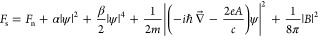
12where *F*_n_ denotes the normal free energy of the system (not in the superconducting state) and *m* describes the mass of the electron. It becomes clearer later with BCS theory why the charge is 2*e* (Cooper pair) instead of *e* (a single electron). *A* is the vector potential, and *B* is the magnetic field.

Using the Helmholtz free energy equation, one may now continue to determine the other variables, where *H*_c_ denotes the magnitude of the energy needed to keep the magnetic field outside the material.^106^
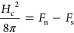
13In the absence of a magnetic field, the equation simplifies to the first two terms (assuming an equilibrium state) with  being a minimum of free energy. Both α and β can now be readily computed using .^109^
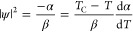
14It was known from experiments that eq 14 corresponds to the magnetic field *H*^2^. Again, Ginzburg and Landau minimized the free energy *F*_s_ by taking the derivative  and applying the boundary condition that neither the gradient of ψ nor the direction of the vector potential acting on ψ has parallel components at the boundary, which they call the natural boundary condition.

Again minimizing the free energy (with respect to *A* and ψ, but this time using eq 12), one may now derive the two Ginzburg–Landau equations.^106,109^
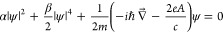
15and
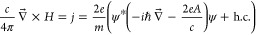
16When using a hydrodynamic model to describe charge transport in an SC, one may now deduce more meaning from the wave-like function ψ. We can relate this variable to the number density: *n*(*r*) = |ψ(*r*)|^2^ with the current equation *j* = 2*env*^106^ or more precisely to the density matrix ρ = *∫n*(*r*′) d*r*′.^109^

That leads us right to the SC’s charge velocity:
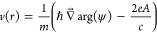
17This approach further enhanced the understanding of the macroscopic behavior of SCs such as the previously described type-II SC. Using the Ginzburg–Landau theory, Abrikosov introduced magnetic field vertices that entered the solid, thus allowing for mixed state SCs.^110^ This mixed state entailed that the material would be neither a strictly superconducting nor a normal material.

#### BCS Theory

While Ginzburg and Landau had focused heavily on the phenomenological description of superconductivity, Bardeen, Cooper, and Schrieffer managed to describe the superconducting state on the microscopic level. They argued that, given low temperatures, electrons (spin 1/2) could pair up to so-called Cooper pairs and form quasi-bosons (energy particles). This electron pair wave function could then travel through matter freely, as low temperatures (low energy states) annihilate electron–proton scattering effects.^102^

Later called the BCS theory, in 1957 Bardeen, Cooper, and Schrieffer sought to explain the second order phase transition at the critical temperature *T*_C_, the experimental measurement of energy gaps for the electron excitations, the Meissner–Ochsenfeld effect, infinite conductivity, and the dependence on the isotope. They built upon the Bloch model of conductivity and included phonon–electron interaction as well as Coulomb forces to their description of superconductivity, as already proposed by Fröhlich.^111^ They came to the conclusion that thermal electron scattering needs to be addressed in order to theoretically describe these phenomena. In phonon–electron interaction, they found electrons to be attracted to one another if the difference between the electron states (Δϵ) was less than *ℏω*: Δϵ < *ℏω*. Bardeen, Cooper, and Schrieffer thus explained many already discovered physical concepts theoretically and finally received the Nobel Prize for their groundbreaking work in 1972.^102,112^ Gor’kov later derived the macroscopic Ginzburg–Landau theory from BSC theory in 1959.^113^

In terms of the description of unconventional (meaning Cooper) pairing outside of phonon exchange or non-BCS SCs (USCs), many attempts and various solutions are at hand. However, theoreticians’ computations for USCs (including TSCs) have largely been meager. The race for an adequate description of existing USCs is therefore still ongoing.^114^

### Superconducting Energy Gap

One of the most central quantities in the description of conventional SCs, especially for the experimental confirmation of superconductivity, is the superconducting energy gap Δ in the energy dispersion relation, which results from the Cooper pairing mechanism, as the electrons condense into the BCS ground state. They are thus no longer available to the fermionic energy spectrum.^115^ To measure this temperature-dependent quantity, one oftentimes uses angle-resolved photoemission spectroscopy (ARPES) or scanning tunneling microscopy (STM) (examples: refs (116, 117, 118)), which have become standard experimental condensed matter physics tools.^78,119^ The gap may also be measured directly, as demonstrated by Nicol et al. in 1960.^120^ One may also use heat capacity measurements to deduce Δ.^115^

Once Δ has been determined, one may use it to fit it to BCS theory to yield characteristic superconducting phase transition diagrams.^116^

### Post-BCS Developments

Along with the first real-world applications of early superconductors, soon new kinds of materials became of interest to the scientific community: partially filled 3d, 4f, and 5f electronic shells and their chemical compounds in the 1960s. To describe it in short, strongly correlated materials (SCMs).

Initially, the most fascinating phenomenon in these novel materials was the metal–insulator phase transition within the group of transition metal oxides. Thereafter, heavy fermion systems (the effective mass being greater than the free electron’s mass by some factor of hundreds to thousands) were discovered in the 1970s, which yielded interesting phenomena of phase transitions between magnetic moments and superconductivity as well as anomalous transport properties.^121^ One such heavy fermion SC is CeCu_2_Si_2_ with *T*_C_ = 0.79 K discovered by Steglich et al. in 1979.^122^ In opposition to these strongly correlated systems, weak electron correlation may be found in classical band theory (semiconductors, simple metals), in which electron interaction is practically neglected.^121^

Theoretical models of these phenomena were necessary. An early approach was taken by the Englishman John Hubbard: his assumptions neglected electrons of the s and p shells, saying that they may be considered stationary around the nucleus, and only took into account the valence electrons of a given lattice atom. Furthermore, his theory only held for low temperatures (stationary lattice points).^123^ His method stems from the Slater–Koster tight-binding model of solids, which approximates that the electrons of a lattice point are confined within a small region around the atomic nucleus, thus minimally changing the wave function of the electron upon very limited interaction with other lattice points.^124^

An unique event was marked in 1986 by Bednorz and Müller by the discovery of a extraordinarily high *T*_C_ of a cuprate.^125^ They detected a *T*_C_ of around 30 K. At first, the scientific community was sceptical of their results; however, their experiments were confirmedly reproducible by other groups globally within several weeks. Some of them reported even higher *T*_C_ values between 40 and 80 K. Bednorz and Müller were awarded the Nobel Prize in Physics in 1987, just a year later.^126^ Today, the highest measured *T*_C_ of an SC was measured to be 133 K at atmospheric pressure^127^ and 164 K at high pressure (31 GPa).^128^

New classes of SCs emerged in the late 2000s: the existence of iron-based SCs was verified in LaOFeAs by Hosono et. al with *T*_C_ = 26 K.^129^ While the class of iron-based SCs has grown steadily,^130−132^ around 10 years later, iron-free Pd-based SCs emerged in highly pressurized systems.^133^ Almost simultaneously, a new conventional class of SCs became of interest, namely, hydride SCs. High phonon frequencies, strong electron–phonon coupling, and a high density of states are a promising recipe. Fulfilling these requirements, looking at hydrides seems like a sound path to take,^134^ as shown in a sulfur hydride system with *T*_C_ = 203 K at high pressures.^135^ Many new room temperature, high pressure hydride SCs have been predicted in the last few years.^136−138^

### Early Applications

While superconducting electromagnets do not purely retort the history of superconductivity itself, a small detour into that field shall be made, since today’s commercial superconductors mainly arise in the form of superconducting electromagnets (such as magnetic resonance spectroscopy^139^ and magnetic levitation trains).^140^ While research further continued in the ever-growing field of superconductivity, superconducting materials became readily available, and more and more scientists became invested in superconducting electromagnets.^95^ Early attempts to fabricate large electromagnets were however unsuccessful. Superconducting wires were wound into coils. If the coils, however, scaled too large, the superconducting wire’s ohmic resistance rose such that the temperature of the wire rose. Hence, the coil underperformed compared to the scientists’ expectations. The heating process could be avoided by a loop of cooling the system with liquefied helium again and again, called training.^95^ Since the process was largely unpredictable,^141^ a new method was necessary. Stekly and Zar were able to stabilize such superconductor coils. In 1965, they found that if the initial current carrying capacity of the superconductor exceeded its material-dependent maximum, it would “spill over” into the cooling substrate (helium). Furthermore, they were able to annihilate disturbances by introducing additional copper wires into the coil. If a disturbance appeared (magnetic flux, etc.) and part of the material returned from the superconducting to the normal state, these copper wires would conduct the current until the superconducting material recovered to its superconducting state. This detour of the current is necessary, as the normal conduction zone of the superconductor cannot recover on its own. This is due to the reduction in conductivity leading to a further disturbance in the form of an increase in heat output.^141^ In the early stages, such superconducting coils were a main interest for early stage particle accelerators.^142^

## Topological Superconductors (TSCs)

The field of topological superconductivity is particularly interesting since it combines two fields of research: TMs and SCs. However, TSCs do not act according to the BCS theory. A regular SC has an adiabatic (smooth) transition between the BCS and the Bose–Einstein condensate of Cooper pairs, which may be tuned by an interaction strength parameter. As discussed in TMs, we allow no adiabatic transition between the two topologically different states. Hence, the classic theory of superconductivity does not describe TSCs well enough, and we need to look into more unconventional pairing mechanisms.^8^

If the BCS theory does not hold, how does a TM become superconducting? The answer lies in the protected gapless excitations at the boundary of the material. Gapless means that if the momentum *k* → 0, then the energy *E* → 0. It gets even more interesting if we consider that both time-reversal symmetry breaking TSCs (closely related to quantum Hall insulators) and time-reversal invariant TSCs (related to topological insulators) exist.^8^ The mentioned gapless excitations, however, do not stem from typical electron/hole mechanisms but are instead electron/hole superpositions of Bogoliubov quasi-particles,^8^ often referred to as Majorana fermions.^143^ Conceptually, they may be described as being their own antiparticle in an excited state. In easier terms, they are their own hole.^144^ Since Majorana fermions must have the same properties as their antiparticles, they may not possess electric charge or spin. Finally, odd-parity pairing of the electron wave functions is essential.^25^ Thus, three requirements have to be fulfilled: first, we require odd-parity pairing symmetry with a superconducting gap. Additionally, we need gapless surface states consisting of Majorana fermions, and finally, we require MZMs in the superconducting vortex cores.^25^

As mentioned in the previous paragraph, some TSCs exhibit time-reversal symmetry breaking, while others do not. We shall investigate how these two distinct TMs obtain their superconducting phase and name some real materials exhibiting such phases, to which one may refer. For the discussion of the TRI and TR SCs, we will refer to 2D systems, as a generalization can be made in a straightforward fashion, as in ref (145).

### Classification of Topological Superconductors by Time-Reversal Symmetry

**Figure 8:**
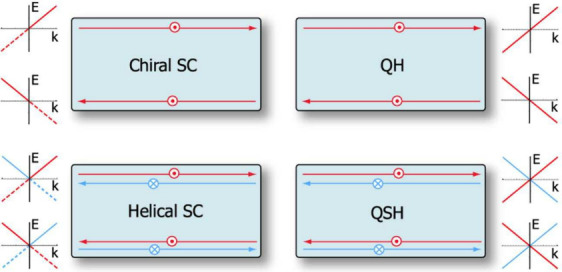
Schematic of electron transport and dispersion relations of chiral and helical SCs opposing their nonsuperconducting quantum Hall and quantum spin Hall relatives, including their dispersion relations. Reprinted with permission from ref (145). Copyright 2009, American Physical Society.

As already mentioned previously, time-reversal symmetry breaking TSCs exhibit quantum Hall-like chiral edge states.^145^ As shown in Figure 8, time-reversal invariant TSCs show similar behavior as in QSHE. The quasi-particle responsible for electron transport in topological insulators is the Dirac fermion, following the Dirac equation and being massless.^145,146^ The quasi-particle underlying TRI SCs are helical chiral Majorana fermions, only having half the degrees of freedom as the Dirac fermions.^147^ TRI SCs also channel up and down spins separately, opposing TRS breaking SCs, as can be seen in the figure.

Let us now consider the dispersion relations of the four materials at hand. The nonsuperconducting states (QHE and QSHE) show the spin-channel behavior well. Looking at the top right dispersion relation, it becomes clear that upon a TR operator acting on the state/system, the spins flip and the direction of momenta *k* do as well, yielding the expected outcome: a linear dispersion relation is reflected in the *k*-plane. Similarly, but with separate spin channels (red and blue), the dispersion relation of the QSHE is also reflected but with no overall change of the dispersion relation since a reflection in the *k*-plane retains the same dispersion.

The superconducting dispersion relations (very left of Figure 8) behave similarly but only allow for positive energy values. For a detailed derivation from the Bogoliubov–de Gennes Hamiltonian in the Nambu basis to the *E* = −*E* and *k* = −*k* equality in the dispersion relation of Majorana modes, the reader is advised to study refs (147) and (7). In short, the reason for that phenomenon is that Majorana fermions are Kramers degenerate. If a TR operator *T* acts on a state |ψ⟩, the eigenenergy of the system remains the same.^148^ Even though, in general, their  spin of electrons forbids the same quantum state, one should recall that they are occupied with Majorana fermions, making the negative energies redundant, as positive and negative solutions may be computed using the same *k*-values.^145^ Majorana fermions do not depend on momenta greater than zero.^7^

### Classification by the Coupling Strength and Pairing Mechanism

Yet another distinction of TSCs can be made by means of their coupling strengths and the Cooper paring mechanism. As already described in a previous section, the main goal to achieve superconductivity is to find some shielding mechanisms (often referred to as screening) that make originally repulsive electrons attract each other in some channel to form Cooper pairs. This is usually done via some angular momentum channel.^149^ Already in the 1960s, Kohn and Luttinger concluded that, in principle, superconductivity could endure even at minimal coupling strengths in a 3D electron gas with short interaction lengths.^149,150^ This was later confirmed in numerous unconventional SCs like cuprate SC and organic SC.^149^

A strongly coupled SC (SCS), examples being Pb and Hg,^151^ on the other hand cannot be described in the same fashion. A weakly coupled SC (WCS), examples being Al and Zn,^151^ may be described using Landau’s quasi-particle approximation,^152^ in which the strongly interacting electrons are replaced by weakly interacting quasi-particles near the Fermi surface.^153^ Within an SCS, Landau’s quasi-particle approximation breaks down^151^ and has to be replaced by a formulation of Eliashberg, in which Green’s functions for electrons in an SC computed by Gor’kov come into play.^154^ A classification via coupling should not be confused by one via correlation strengths, which is, of course, also possible. An example of a strongly correlated SC is Rh_17_S_15_,^155^ while Mo_3_Al_2_C^156^ is considered a weakly correlated SCs.

Despite such great success in both theory and experiments, when looking for TSCs, one should not be too concerned with such conventional categorizations since the mechanisms at play are clearly not topological: the material transitions from a weakly coupled BCS quantum state to a strongly coupled Bose–Einstein condensate.^25^ Therefore, we are on the lookout for systems with unconventional electron–electron pairing mechanisms.^25^ Especially, the combination of TRS breaking and spin-triplet pairing in materials has promised more strong coupling TSCs. To this day, however, few materials of the kind exist.^25^

TSCs may exhibit weak and strong electron–phonon and electron–electron coupling.^25,149^ An example of a strong coupling TSC is the doped semimetal Mo_5_Si_3_,^157^ whereas Sr_2_RuO_4_ is a good example of a weak coupling TSC.^149^

Not only the coupling strength but also the coupling mechanism should be of interest when concerned with a TSC.^25^ Conventional SC Cooper pairs usually pair via the s- or d-wave (*L* = 0 and 2, respectively). The total spin is *S* = 0, a singlet. TSC pairing happens via triplet (*S* = 1) pairing, which leads to p-, f-, and h-wave superconductivity, because the overall wave function must be odd parity.^25^ A prominent example of such an odd-parity TSC is Cu_*x*_Bi_2_Se_3_, which has received considerable attention in the previous decade.^158−160^ A more detailed classification of bulk SCs may be found in ref (160).

## Future Perspectives and Open Questions

As the exploration of topological quantum materials continues to advance, several promising avenues emerge for future research. One key area is the investigation of novel topological superconductors that exhibit unconventional pairing mechanisms, which could host Majorana fermions—quasi-particles with potential applications in fault-tolerant quantum computing. Understanding the precise conditions and material properties that facilitate the emergence of these exotic states remains a critical challenge. Additionally, integrating topological quantum materials with existing semiconductor technologies could pave the way for hybrid devices that leverage both topological protection and conventional electronic functionalities, fostering innovations in spintronics and quantum information processing.

Despite significant progress, numerous open questions persist in the study of the interplay between superconductivity and other electronic orders in topological materials. For instance, the mechanisms governing the coexistence or competition between superconductivity and magnetism in these systems are not yet fully understood. Furthermore, the role of electron correlations and disorder in shaping the topological and superconducting properties warrants a deeper investigation. Addressing these questions requires a multidisciplinary approach, combining advanced theoretical models with cutting-edge experimental techniques, such as scanning tunneling microscopy and angle-resolved photoemission spectroscopy. By tackling these challenges, researchers can unlock new fundamental insights and drive the development of next-generation quantum technologies.

A comparative analysis of different topological superconductors and their unique properties could provide deeper insights into their respective advantages and challenges. By systematically evaluating various classes of topological superconductors, such as those based on iron-based compounds, heavy fibril systems, and engineered heterostructures, researchers can identify common mechanisms that facilitate topological superconductivity as well as distinct pathways that lead to diverse behaviors. This comparison can elucidate factors that enhance stability, coherence, and the emergence of exotic quasi-particles, such as Majorana fermions, which are pivotal for quantum computing applications. Additionally, understanding the limitations and practical challenges associated with each type, such as material synthesis difficulties, temperature constraints, and sensitivity to disorder, can guide the development of more robust and scalable topological superconducting systems. Ultimately, this comparative approach will not only advance fundamental knowledge but also inform the design of next-generation quantum devices with optimized performance and functionality.

## Conclusion and Outlook

In conclusion, topological quantum materials have emerged as a fascinating and rapidly developing field of research at the intersection of condensed matter physics and materials science. These materials possess unique electronic properties and exhibit nontrivial topological characteristics that give rise to protected states and novel transport phenomena. The exploration of topological quantum materials has not only deepened our fundamental understanding of condensed matter physics but also opened up promising avenues for technological applications. The study of topological quantum materials has led to the discovery of various intriguing phenomena and the proposal of potential applications. Major advancements have been made in the understanding and manipulation of topological insulators, topological superconductors, and other topological phases. The discovery of Majorana zero modes, topologically protected surface states, and topological edge states has raised excitement for their potential use in topological quantum computing, fault-tolerant quantum gates, and robust information storage. Moreover, the investigation of topological quantum materials has led to the development of new techniques and experimental tools for characterizing and probing these materials. Scanning probe microscopy, angle-resolved photoemission spectroscopy (ARPES), and advanced transport measurements have played pivotal roles in the experimental study of topological quantum materials, allowing for the direct observation and manipulation of their unique electronic properties. Looking forward, the field of topological quantum materials holds great potential for further discoveries and technological breakthroughs. Ongoing research aims to uncover new topological phases, explore their properties in different material systems, and engineer novel functionalities. Additionally, efforts are being made to better understand the interplay of topological physics with other phenomena, such as strong correlations, magnetic order, and unconventional superconductivity. The development of topological quantum materials also benefits from the synergy between theory and experiment. Theoretical models and simulations, along with experimental validation, guide the search for new materials and aid in the understanding of their behavior. This interdisciplinary collaboration continues to drive the field forward and has the potential to lead to transformative advances in areas like quantum computing, spintronics, and energy efficient electronics. The field is still rapidly evolving with ongoing discoveries, theoretical advancements, and experimental breakthroughs. The future of topological quantum materials is filled with excitement and the potential to reshape our technological landscape.

In a nutshell, we discussed the main causes of topological phenomena. The most fundamental mathematical tools to describe topological materials such as the Chern number, the principal concepts of time and parity symmetry, and MZM were explained. With the attained knowledge, we were then able to understand the integer QHE as well as the related effects, including the QAHE and QSHE but also FQHE. We then deepened our knowledge on numerous topological materials. They often relate to the various Hall-related effects, for example, the QSHE in TIs. Furthermore, relatively unexplored and novel materials such as CCs and MTIs became of substantial interest.

In the second part, we took a historical perspective on superconductivity, from Kamerlingh Onnes discovery^93^ more than a hundred years ago to today’s ongoing research in high pressure hydrides.^135^ We looked at the classification between type-I and type-II SCs. Besides the experimental findings by Kamerlingh Onnes^93^ and the Meissner–Ochsenfeld effect,^98^ ground-laying theoretical work such as the London equations^103^ and Ginzburg–Landau theory^106^ was discussed in brief, followed by a short introduction to complete the description of conventional superconductivity by Bardeen, Cooper, and Schrieffer.^102^ Finally, the fields of topology were brought into contact with superconductivity to form TSCs. TSCs were then classified by their TRS, coupling strength, and pairing mechanism to give a better overview of that quickly emerging field.

With large databases and advanced methods such as DFT, it has become easier to find TSCs than previously possible by means of symmetry indicators. Such symmetry indicators may be found in the electronic structure of the material to detect nontrivial topological phases.^161^ New types of TSCs are also popping up in various places. Examples include superconducting WSMs,^47^ nodal TSCs driven by magnetic fields,^162^ and artificial TSCs caused by moiré patterns.^162^ Not only by their various foundations but also by their synthetization method, advancements are to be expected: hard tip point contact method, intercalation , doping, and electric field gating are again only examples of how topological materials and more specifically TSCs may be realized.^25^ Current topics of research include raising temperatures, ultrafast studies, exotic Fermi arc behavior,^163^ CCs with magnonic systems,^164^ optical response behavior, and the Jahn–Teller effect, which can help to stabilize MTIs.^165^

While the main focus of applying TSCs is clearly quantum computing,^25^ topological materials may become appealing to many different branches of engineering due to their low power dissipation. Examples include catalysis, energy conversion, data and energy storage, spintronics devices and metrology.^33,34,37,166,167^ However, the number of applications that these interesting materials shall have in the future, only time will tell.

## References

[ref1] Kosterlitz J. M. Kosterlitz–Thouless physics: a review of key issues. Rep. Prog. Phys. 2016, 79, 026001 10.1088/0034-4885/79/2/026001. 26824490

[ref2] Wieder B. J.; Bradlyn B.; Cano J.; Wang Z.; Vergniory M. G.; Elcoro L.; Soluyanov A. A.; Felser C.; Neupert T.; Regnault N.; Bernevig B. A. Topological materials discovery from crystal symmetry. Nature Reviews Materials 2022, 7, 196 10.1038/s41578-021-00380-2.

[ref3] Kane C. L.; Mele E. J. Quantum spin Hall effect in graphene. Physical review letters 2005, 95, 226801 10.1103/PhysRevLett.95.226801. 16384250

[ref4] Menth A.; Buehler E.; Geballe T. H. Magnetic and semiconducting properties of Sm B 6. Phys. Rev. Lett. 1969, 22, 295–297. 10.1103/PhysRevLett.22.295.

[ref5] Crassee I.; Sankar R.; Lee W.; Akrap A.; Orlita M. 3D Dirac semimetal Cd 3 As 2: A review of material properties. Physical Review Materials 2018, 2, 120302 10.1103/PhysRevMaterials.2.120302.

[ref6] von Klitzing K. The quantized Hall effect. Rev. Mod. Phys. 1986, 58, 519–538. 10.1103/RevModPhys.58.519.

[ref7] Bernevig B. A. In Topological insulators and topological superconductors; Princeton University Press, 2013.

[ref8] Ando Y.; Fu L. Topological Crystalline Insulators and Topological Superconductors: From Concepts to Materials. Annual Review of Condensed Matter Physics 2015, 6, 361–381. 10.1146/annurev-conmatphys-031214-014501.

[ref9] Qi X.-L.; Zhang S.-C. Topological insulators and superconductors. Rev. Mod. Phys. 2011, 83, 1057 10.1103/RevModPhys.83.1057.

[ref10] Chang G.; Wieder B. J.; Schindler F.; Sanchez D. S.; Belopolski I.; Huang S.-M.; Singh B.; Wu D.; Chang T.-R.; Neupert T.; Xu S.-Y.; Lin H.; Hasan M. Z. Topological quantum properties of chiral crystals. Nature Materials 2018, 17, 978–985. 10.1038/s41563-018-0169-3. 30275564

[ref11] Yan B.; Zhang S.-C. Topological materials. Rep. Prog. Phys. 2012, 75, 096501 10.1088/0034-4885/75/9/096501. 22907264

[ref12] Liu P.; Williams J. R.; Cha J. J. Topological nanomaterials. Nature Reviews Materials 2019, 4, 479–496. 10.1038/s41578-019-0113-4.

[ref13] Yoshioka D. In The Quantum Hall Effect; Springer, Berlin, Heidelberg, 2002.

[ref14] Advances in Topological Materials; Pronin A., Ed.; MDPI, 2022.

[ref15] Weng H.; Yu R.; Hu X.; Dai X.; Fang X. Quantum anomalous Hall effect and related topological electronic states. Adv. Phys. 2015, 64, 227–282. 10.1080/00018732.2015.1068524.

[ref16] Stormer H. L.; Tsui D. C.; Gossard A. C. The fractional quantum Hall effect. Rev. Mod. Phys. 1999, 71, S298 10.1103/RevModPhys.71.S298.

[ref17] Yu R.; Zhang W.; Zhang H.-J.; Zhang S.-C.; Dai X.; Fang Z. Quantized anomalous Hall effect in magnetic topological insulators. science 2010, 329, 61–64. 10.1126/science.1187485. 20522741

[ref18] Fei F.; Zhang S.; Zhang M.; Shah S. A.; Song F.; Wang X.; Wang B. The Material Efforts for Quantized Hall Devices Based on Topological Insulators. Adv. Mater. 2020, 32, 1904593 10.1002/adma.201904593. 31840308

[ref19] Armitage N.; Mele E.; Vishwanath A. Weyl and Dirac semimetals in three-dimensional solids. Rev. Mod. Phys. 2018, 90, 015001 10.1103/RevModPhys.90.015001.

[ref20] He K. The quantum Hall effect gets more practical. Physics 2015, 8, 41 10.1103/Physics.8.41.

[ref21] Konig M.; Wiedmann S.; Brune C.; Roth A.; Buhmann H.; Molenkamp L. W.; Qi X.-L.; Zhang S.-C. Quantum spin Hall insulator state in HgTe quantum wells. Science 2007, 318, 766–770. 10.1126/science.1148047. 17885096

[ref22] Chang G.; Xu S.-Y.; Huang S.-M.; Sanchez D. S.; Hsu C.-H.; Bian G.; Yu Z.-M.; Belopolski I.; Alidoust N.; Zheng H.; Chang T.-R.; Jeng H.-T.; Yang S. A.; Neupert T.; Lin H.; Hasan M. Z. Nexus fermions in topological symmorphic crystalline metals. Sci. Rep. 2017, 7, 1688 10.1038/s41598-017-01523-8. 28490762 PMC5431971

[ref23] Wang P.; Ge J.; Li J.; Liu Y.; Xu Y.; Wang J. Intrinsic magnetic topological insulators. Innovation 2021, 2, 100098 10.1016/j.xinn.2021.100098. 34557750 PMC8454723

[ref24] Laughlin R. B. Quantized Hall conductivity in two dimensions. Phys. Rev. B 1981, 23, 5632 10.1103/PhysRevB.23.5632.

[ref25] Li Y.; Xu Z.-A. Exploring topological superconductivity in topological materials. Advanced Quantum Technologies 2019, 2, 1800112 10.1002/qute.201800112.

[ref26] Weng H.; Fang C.; Fang Z.; Bernevig B. A.; Dai X. Weyl semimetal phase in noncentrosymmetric transition-metal monophosphides. Physical Review X 2015, 5, 011029 10.1103/PhysRevX.5.011029.

[ref27] Han J.; Liu L. Topological insulators for efficient spin–orbit torques. APL Materials 2021, 9, 060901 10.1063/5.0048619.

[ref28] Sau J. D.; Lutchyn R. M.; Tewari S.; Das Sarma S. Generic new platform for topological quantum computation using semiconductor heterostructures. Physical review letters 2010, 104, 040502 10.1103/PhysRevLett.104.040502. 20366693

[ref29] Appugliese F.; Enkner J.; Paravicini-Bagliani G. L.; Beck M.; Reichl C.; Wegscheider W.; Scalari G.; Ciuti C.; Faist J. Breakdown of topological protection by cavity vacuum fields in the integer quantum Hall effect. Science 2022, 375, 1030–1034. 10.1126/science.abl5818. 35239382

[ref30] Schreiber K. A. The Quantum Hall Effect. In: Ground States of the Two-Dimensional Electron System at Half- Filling Under Hydrostatic PressureFilling Under Hydrostatic Pressure. Springer Theses. Springer, Cham, 2018. 10.1007/978-3-030-26322-5_1.

[ref31] Lau A. Symmetry-enriched topological states of matter in insulators and semimetals. Ph.D. Dissertation, Technische Universität Dresden, 2018.

[ref32] Hasan M. Z.; Kane C. L. Colloquium: Topological insulators. Rev. Mod. Phys. 2010, 82, 3045–3067. 10.1103/RevModPhys.82.3045.

[ref33] Gilbert M. J. Topological electronics. Communications Physics 2021, 4, 70 10.1038/s42005-021-00569-5.

[ref34] Poirier W.; Schopfer F. Resistance metrology based on the quantum Hall effect. European Physical Journal Special Topics 2009, 172, 207–245. 10.1140/epjst/e2009-01051-5.

[ref35] He Q. L.; Hughes T. L.; Armitage N. P.; Tokura Y.; Wang K. L. Topological spintronics and magnetoelectronics. Nature materials 2022, 21, 15–23. 10.1038/s41563-021-01138-5. 34949869

[ref36] Li G.; Felser C. Heterogeneous catalysis at the surface of topological materials. Appl. Phys. Lett. 2020, 116, 070501 10.1063/1.5143800.

[ref37] Luo H.; Yu P.; Li G.; Yan K. Topological quantum materials for energy conversion and storage. Nature Reviews Physics 2022, 4, 611–624. 10.1038/s42254-022-00477-9.

[ref38] Budich J. C.; Bergholtz E. J. Non-Hermitian topological sensors. Phys. Rev. Lett. 2020, 125, 180403 10.1103/PhysRevLett.125.180403. 33196268

[ref39] Moore J. E. The birth of topological insulators. Nature 2010, 464, 194–198. 10.1038/nature08916. 20220837

[ref40] Maeno Y.; Hashimoto H.; Yoshida K.; Nishizaki S.; Fujita T.; Bednorz J.; Lichtenberg F. Superconductivity in a layered perovskite without copper. nature 1994, 372, 532–534. 10.1038/372532a0.

[ref41] Sato M.; Ando Y. Topological superconductors: a review. Rep. Prog. Phys. 2017, 80, 076501 10.1088/1361-6633/aa6ac7. 28367833

[ref42] Imai Y.; Wakabayashi K.; Sigrist M. Topological aspect and transport property in multi-band spin-triplet chiral p-wave superconductor Sr2RuO4. Journal of Physics: Conference Series. 2015, 592, 012132 10.1088/1742-6596/592/1/012132.

[ref43] Pollmann F.; Schnyder A. Klassifizierung symmetriegeschützter topologischer Phasen. Physik Journal 2015, 14, 65–69.

[ref44] Rohrlich D. Berry’s phase. In Compendium of Quantum Physics; Springer, 2009; p 31–36.

[ref45] Kane C. L. Topological Band Theory and the Z_2_ Invariant. In Contemporary Concepts of Condensed Matter Science; Elsevier, 2013; Vol. 6; p 3–34.

[ref46] Schüler M.; De Giovannini U.; Hübener H.; Rubio A.; Sentef M. A.; Werner P. Local Berry curvature signatures in dichroic angle-resolved photoelectron spectroscopy from two-dimensional materials. Science advances 2020, 6, eaay2730 10.1126/sciadv.aay2730. 32158939 PMC7048418

[ref47] Yan B.; Felser C. Topological materials: Weyl semimetals. Annual Review of Condensed Matter Physics 2017, 8, 337–354. 10.1146/annurev-conmatphys-031016-025458.

[ref48] Maciejko J.; Hughes T. L.; Zhang S.-C. The quantum spin Hall effect. Annu. Rev. Condens. Matter Phys. 2011, 2, 31–53. 10.1146/annurev-conmatphys-062910-140538.

[ref49] Henley E. M. Parity and time-reversal invariance in nuclear physics. Annual Review of Nuclear Science 1969, 19, 367–432. 10.1146/annurev.ns.19.120169.002055.

[ref50] Fano U.; Rau A. R. P. In Symmetries in Quantum Physics; Elsevier, 1996.

[ref51] Turner A. M.; Pollmann F.; Berg E. Topological phases of one-dimensional fermions: An entanglement point of view. Physical review b 2011, 83, 075102 10.1103/PhysRevB.83.075102.

[ref52] Barron L. D.; Buckingham A. D. Time Reversal and Molecular Properties. Acc. Chem. Res. 2001, 34, 781–789. 10.1021/ar0100576. 11601962

[ref53] Bernevig B. A.; Hughes T. L.; Zhang S.-C. Quantum spin Hall effect and topological phase transition in HgTe quantum wells. science 2006, 314, 1757–1761. 10.1126/science.1133734. 17170299

[ref54] Thouless D. J.; Kohmoto M.; Nightingale M. P.; den Nijs M. Quantized Hall conductance in a two-dimensional periodic potential. Physical review letters 1982, 49, 405–408. 10.1103/PhysRevLett.49.405.

[ref55] Majorana E. Teoria simmetrica dell’elettrone e del positrone. Nuovo Cim 1937, 14, 171–184. 10.1007/BF02961314.

[ref56] Jäck B.; Xie Y.; Yazdani A. Detecting and distinguishing Majorana zero modes with the scanning tunnelling microscope. Nature Reviews Physics 2021, 3, 541–554. 10.1038/s42254-021-00328-z.

[ref57] Black-Schaffer A. M.; Linder J. Majorana fermions in spin-orbit-coupled ferromagnetic Josephson junctions. Phys. Rev. B 2011, 84, 180509 10.1103/PhysRevB.84.180509.

[ref58] Akhmerov A. R.; Nilsson J.; Beenakker C. W. J. Electrically detected interferometry of Majorana fermions in a topological insulator. Physical review letters 2009, 102, 216404 10.1103/PhysRevLett.102.216404. 19519120

[ref59] Hall E. H. On a new action of the magnet on electric currents. American Journal of Mathematics 1879, 2 (2), 287–292. 10.2307/2369245.

[ref60] Landau L. Diamagnetismus der metalle. Zeitschrift für Physik 1930, 64, 629–637. 10.1007/BF01397213.

[ref61] Yang C. H.; Peeters F. M.; Xu W. Landau-level broadening due to electron-impurity interaction in graphene in strong magnetic fields. Phys. Rev. B 2010, 82, 075401 10.1103/PhysRevB.82.075401.

[ref62] Reis M. In Fundamentals of magnetism; Elsevier, 2013.

[ref63] Liu C.-X.; Zhang S.-C.; Qi X.-L. The quantum anomalous Hall effect: theory and experiment. Annual Review of Condensed Matter Physics 2016, 7, 301–321. 10.1146/annurev-conmatphys-031115-011417.

[ref64] Pariari A. K. Atoms to topological electronic materials: a bedtime story for beginners. Indian Journal of Physics 2021, 95, 2639 10.1007/s12648-020-01925-x.

[ref65] Chang C.-Z.; Li M. Quantum anomalous Hall effect in time-reversal-symmetry breaking topological insulators. J. Phys.: Condens. Matter 2016, 28, 123002 10.1088/0953-8984/28/12/123002. 26934535

[ref66] Kane C. L.; Mele E. J. Z. 2 topological order and the quantum spin Hall effect. Physical review letters 2005, 95, 146802 10.1103/PhysRevLett.95.146802. 16241681

[ref67] Schwarzschild B. Physics Nobel Prize goes to Tsui, Stormer and Laughlin for the fractional Quantum Hall effect. Phys. Today 1998, 51, 17–19. 10.1063/1.882480.

[ref68] Molecular Beam Epitaxy: From Research to Mass Production; Henini M., Ed.; Elsevier, 2012.

[ref69] Laughlin R. B. Anomalous quantum Hall effect: an incompressible quantum fluid with fractionally charged excitations. Phys. Rev. Lett. 1983, 50, 1395 10.1103/PhysRevLett.50.1395.

[ref70] Feldman D. E.; Halperin B. I. Fractional charge and fractional statistics in the quantum Hall effects. Rep. Prog. Phys. 2021, 84, 076501 10.1088/1361-6633/ac03aa. 34015771

[ref71] Lin X.; Du R.; Xie X. Recent experimental progress of fractional quantum Hall effect: 5/2 filling state and graphene. National Science Review 2014, 1, 564–579. 10.1093/nsr/nwu071.

[ref72] Wang C.; Gioia L.; Burkov A. A. Fractional quantum Hall effect in Weyl semimetals. Phys. Rev. Lett. 2020, 124, 096603 10.1103/PhysRevLett.124.096603. 32202893

[ref73] Thomas L. H. The motion of the spinning electron. Nature 1926, 117, 514–514. 10.1038/117514a0.

[ref74] Hsieh D.; Qian D.; Wray L.; Xia Y.; Hor Y. S.; Cava R. J.; Hasan M. Z. A topological Dirac insulator in a quantum spin Hall phase. Nature 2008, 452, 970–974. 10.1038/nature06843. 18432240

[ref75] Dzero M.; Xia J.; Galitski V.; Coleman P. Topological kondo insulators. Annual Review of Condensed Matter Physics 2016, 7, 249–280. 10.1146/annurev-conmatphys-031214-014749.

[ref76] Dirac P. A. M. The quantum theory of the electron. Proc. R. Soc. Lond. A 1928, 117, 610–624. 10.1098/rspa.1928.0023.

[ref77] Weyl H. Electron and gravitation. z. Phys. 1929, 56, 330–352. 10.1007/BF01339504.

[ref78] Zhang Y.; Tan Y.; Stormer H. L.; Kim P. Experimental observation of the quantum Hall effect and Berry’s phase in graphene. Nature 2005, 438, 201–204. 10.1038/nature04235. 16281031

[ref79] Wang Z.; Weng H.; Wu Q.; Dai X.; Fang Z. Three-dimensional Dirac semimetal and quantum transport in Cd_3_As_2_. Phys. Rev. B 2013, 88, 125427 10.1103/PhysRevB.88.125427.

[ref80] Kane E. O. Band structure of indium antimonide. J. Phys. Chem. Solids 1957, 1, 249–261. 10.1016/0022-3697(57)90013-6.

[ref81] Rosenman I. Effet Shubnikov de Haas dans Cd3As2: Forme de la surface de Fermi et modele non parabolique de la bande de conduction. J. Phys. Chem. Solids 1969, 30, 1385–1402. 10.1016/0022-3697(69)90200-5.

[ref82] Li H.; Xu S.; Rao Z.-C.; Zhou L.-Q.; Wang Z.-J.; Zhou S.-M.; Tian S.-J.; Gao S.-Y.; Li J.-J.; Huang Y.-B.; Lei H.-C.; Weng H.-M.; Sun Y.-J.; Xia T.-L.; Qian T.; Ding H. Chiral fermion reversal in chiral crystals. Nat. Commun. 2019, 10, 5505 10.1038/s41467-019-13435-4. 31796737 PMC6890713

[ref83] Sergelius P.; Gooth J.; Baßler S.; Zierold R.; Wiegand C.; Niemann A.; Reith H.; Shekhar C.; Felser C.; Yan B.; Nielsch K. Berry phase and band structure analysis of the Weyl semimetal NbP. Sci. Rep. 2016, 6, 33859 10.1038/srep33859. 27667203 PMC5036179

[ref84] Xu S.-Y.; Belopolski I.; Alidoust N.; Neupane M.; Bian G.; Zhang C.; Sankar R.; Chang G.; Yuan Z.; Lee C.-C.; Huang S.-M.; Zheng H.; Ma J.; Sanchez D. S.; Wang B.; Bansil A.; Chou F.; Shibayev P. P.; Lin H.; Jia S.; Hasan M. Z. Discovery of a Weyl fermion semimetal and topological Fermi arcs. Science 2015, 349, 613–617. 10.1126/science.aaa9297. 26184916

[ref85] Nielsen H. B.; Ninomiya M. The Adler-Bell-Jackiw anomaly and Weyl fermions in a crystal. Physics Letters B 1983, 130, 389–396. 10.1016/0370-2693(83)91529-0.

[ref86] Bogdanov A.; Hubert A. Thermodynamically stable magnetic vortex states in magnetic crystals. J. Magn. Magn. Mater. 1994, 138, 255–269. 10.1016/0304-8853(94)90046-9.

[ref87] Kramers H. A. Théorie générale de la rotation paramagnétique dans les cristaux. Proc. Acad. Amst 1930, 33, 959–972.

[ref88] Sessi P.; Fan F.; Küster F.; Manna K.; Schröter N. B. M.; Ji J.; Stolz S.; Krieger J. A.; Pei D.; Kim T. K.; et al. Handedness-dependent quasiparticle interference in the two enantiomers of the topological chiral semimetal PdGa. Nat. Commun. 2020, 11, 3507 10.1038/s41467-020-17261-x. 32665572 PMC7360625

[ref89] Gokhfeld D.; Popkov S.; Bykov A. Analog of the intertype superconductivity in nanostructured materials. Physica C: Superconductivity and its Applications 2019, 566, 1353526 10.1016/j.physc.2019.1353526.

[ref90] Wang J.; Lian B.; Zhang S.-C. Quantum anomalous Hall effect in magnetic topological insulators. Phys. Scr. 2015, T164, 014003 10.1088/0031-8949/2015/T164/014003.

[ref91] Jiang Z.; Liu J.; Liu Z.; Shen D. A review of angle-resolved photoemission spectroscopy study on topological mangetic material family of MnBi2Te4. Electronic Structure 2022, 4 (4), 043002 10.1088/2516-1075/acab47.

[ref92] Chang C.-Z.; Zhang J.; Feng X.; Shen J.; Zhang Z.; Guo M.; Li K.; Ou Y.; Wei P.; Wang L.-L.; et al. Experimental observation of the quantum anomalous Hall effect in a magnetic topological insulator. Science 2013, 340, 167–170. 10.1126/science.1234414. 23493424

[ref93] van Delft D.; Kes P. The discovery of superconductivity. Phys. Today 2010, 63, 38–43. 10.1063/1.3490499.

[ref94] All Nobel Prizes. https://www.nobelprize.org/prizes/lists/all-nobel-prizes/ (accessed 2024-01-15).

[ref95] Wilson M. N. 100 Years of Superconductivity and 50 Years of Superconducting Magnets. IEE Transactions on Applied Superconductivity 2012, 22, 3800212 10.1109/TASC.2011.2174628.

[ref96] de Haas W. J. Newly Discovered Superconductors. Nature 1929, 123, 130–131. 10.1038/123130c0.

[ref97] Shubnikov L. V.; Khotkevich V. I.; Shepelev Y. D.; Ryabinin Y. N. Magnetic properties of superconducting metals and alloys. Zh. Eksp. Teor. Fiz 1937, 7, 221–237.

[ref98] Meissner W.; Ochsenfeld R. Ein neuer Effekt bei Eintritt der Supraleitfähigkeit. Naturwissenschaften 1933, 21 (44), 787 10.1007/BF01504252.

[ref99] Rey C.; Malozemoff A. Fundamentals of Superconductivity. In Superconductors in the Power Grid; Rey C., Ed.; Woodhead Publishing, 2015; p 29–73.

[ref100] Hofmann P. In Solid State Physics: An Introduction; John Wiley & Sons, 2015.

[ref101] Schmalian J. Failed theories of superconductivity. Modern Physics Letters B 2010, 24, 2679–2691. 10.1142/S0217984910025280.

[ref102] Bardeen J.; Cooper L. N.; Schrieffer J. R. Theory of Superconductivity. Phys. Rev. 1957, 108, 1175–1204. 10.1103/PhysRev.108.1175.

[ref103] London F.; London H. The electromagnetic equations of the supraconductor. Proc. R. Soc. Lond. A 1935, 149, 71–88. 10.1098/rspa.1935.0048.

[ref104] Einstein A. The Foundation of the General Theory of Relativity. Annalen Phys. 1916, 354, 769–822. 10.1002/andp.19163540702.

[ref105] Gorter C.; Casimir H. In Archives du Musée Teyler: Serie III, Vol. VIII, Fascicule 1, 1st ed.; Springer, Dordrecht, The Netherlands, 1935.

[ref106] Cyrot M. Ginzburg-Landau theory for superconductors. Rep. Prog. Phys. 1973, 36, 103 10.1088/0034-4885/36/2/001.

[ref107] Schmitt A. In Introduction to Superfluidity; Springer, 2015.

[ref108] Hohenberg P.; Krekhov A. An introduction to the Ginzburg–Landau theory of phase transitions and nonequilibrium patterns. Phys. Rep. 2015, 572, 1–42. 10.1016/j.physrep.2015.01.001.

[ref109] Ginzburg V. L.; Landau L. D. On Superconductivity and Superfluidity. In On Superconductivity and Superfluidity: A Scientific Autobiography; Springer, 2009; p 113–137.

[ref110] Abrikosov A. The magnetic properties of superconducting alloys. J. Phys. Chem. Solids 1957, 2, 199–208. 10.1016/0022-3697(57)90083-5.

[ref111] Fröhlich H. Theory of the superconducting state. I. The ground state at the absolute zero of temperature. Phys. Rev. 1950, 79, 845–856. 10.1103/PhysRev.79.845.

[ref112] Pines D.; Slichter C. P. The 1972 Nobel Prize for Physics. Science 1972, 178, 489–491. 10.1126/science.178.4060.489. 17754376

[ref113] Gor’kov L. P. Microscopic derivation of the Ginzburg-Landau equations in the theory of superconductivity. Sov. Phys. JETP 1959, 9, 1364–1367.

[ref114] Stewart G. R. Unconventional superconductivity. Adv. Phys. 2017, 66, 75–196. 10.1080/00018732.2017.1331615.

[ref115] Hofmann P. In Einführung in die Festkörperphysik; John Wiley & Sons, 2013.

[ref116] Eom D.; Qin S.; Chou M.-Y.; Shih C. K. Persistent superconductivity in ultrathin Pb films: A scanning tunneling spectroscopy study. Physical review letters 2006, 96, 027005 10.1103/PhysRevLett.96.027005. 16486621

[ref117] Rodrigo J.; Suderow H.; Vieira S. On the use of STM superconducting tips at very low temperatures. European Physical Journal B-Condensed Matter and Complex Systems 2004, 40, 483–488. 10.1140/epjb/e2004-00273-y.

[ref118] Kitazawa K.; Sugawara H.; Hasegawa T. On the superconducting gap structure of high-temperature superconductors by STM/STS. Physica C: Superconductivity 1996, 263, 214–217. 10.1016/0921-4534(96)00066-4.

[ref119] Meyer E.; Bennewitz R.; Hug H. J. In Scanning Probe Microscopy: The Lab on a Tip; Springer, 2021; p 13–45.

[ref120] Nicol J.; Shapiro S.; Smith P. Direct measurement of the superconducting energy gap. Phys. Rev. Lett. 1960, 5, 461–464. 10.1103/PhysRevLett.5.461.

[ref121] Anisimov V. I. Electronic structure of strongly correlated materials. AIP Conf. Proc. 2010, 1297, 3–134. 10.1063/1.3518902.

[ref122] Steglich F.; Aarts J.; Bredl C. D.; Lieke W.; Meschede D.; Franz W.; Schäfer H. Superconductivity in the presence of strong pauli paramagnetism: Ce cu 2 si 2. Phys. Rev. Lett. 1979, 43, 1892 10.1103/PhysRevLett.43.1892.

[ref123] Hubbard J. Electron correlations in narrow energy bands. II. The degenerate band case. Proc. R. Soc. Lond. A 1964, 277, 237–259. 10.1098/rspa.1964.0019.

[ref124] Slater J. C.; Koster G. F. Simplified LCAO method for the periodic potential problem. Phys. Rev. 1954, 94, 1498–1524. 10.1103/PhysRev.94.1498.

[ref125] Bednorz J. G.; Müller K. A. Possible high T c superconductivity in the Ba- La- Cu- O system. Zeitschrift für Physik B Condensed Matter 1986, 64, 189–193. 10.1007/BF01303701.

[ref126] Buckel W.; Kleiner R. In Superconductivity: Fundamentals and Applications; John Wiley & Sons, 2008.

[ref127] Schilling A.; Cantoni M.; Guo J.; Ott H. Superconductivity above 130 k in the hg–ba–ca–cu–o system. Nature 1993, 363, 56–58. 10.1038/363056a0.

[ref128] Gao L.; Xue Y.; Chen F.; Xiong Q.; Meng R.; Ramirez D.; Chu C.; Eggert J.; Mao H. Superconductivity up to 164 K in HgBa 2 Ca m- 1 Cu m O 2 m+ 2+ δ (m= 1, 2, and 3) under quasihydrostatic pressures. Phys. Rev. B 1994, 50, 4260 10.1103/PhysRevB.50.4260. 9976724

[ref129] Kamihara Y.; Watanabe T.; Hirano M.; Hosono H. Iron-based layered superconductor La [O1-x F x] FeAs (x= 0.05- 0.12) with T c= 26 K. J. Am. Chem. Soc. 2008, 130, 3296–3297. 10.1021/ja800073m. 18293989

[ref130] Van Gennep D.; Hassan A.; Luo H.; Abdel-Hafiez M. Sharp peak of the critical current density in BaFe_2–*x*_Ni_*x*_As_2_ at optimal composition. Phys. Rev. B 2020, 101, 235163 10.1103/PhysRevB.101.235163.

[ref131] Putilov A. V.; Di Giorgio C.; Vadimov V. L.; Trainer D. J.; Lechner E. M.; Curtis J. L.; Abdel-Hafiez M.; Volkova O. S.; Vasiliev A. N.; Chareev D. A.; Karapetrov G.; Koshelev A. E.; Aladyshkin A. Y.; Mel’nikov A. S.; Iavarone M. Vortex-core properties and vortex-lattice transformation in FeSe. Phys. Rev. B 2019, 99, 144514 10.1103/PhysRevB.99.144514.

[ref132] Loos S.; Gruner D.; Abdel-Hafiez M.; Seidel J.; Hüttl R.; Wolter A. U.; Bohmhammel K.; Mertens F. Heat capacity (Cp) and entropy of olivine-type LiFePO4 in the temperature range (2 to 773)K. J. Chem. Thermodyn. 2015, 85, 77–85. 10.1016/j.jct.2015.01.007.

[ref133] Abdel-Hafiez M.; Zhao Y.; Huang Z.; Cho C.-w.; Wong C. H.; Hassen A.; Ohkuma M.; Fang Y.-W.; Pan B.-J.; Ren Z.-A.; Sadakov A.; Usoltsev A.; Pudalov V.; Mito M.; Lortz R.; Krellner C.; Yang W. High-pressure effects on isotropic superconductivity in the iron-free layered pnictide superconductor BaPd2As2. Phys. Rev. B 2018, 97, 134508 10.1103/PhysRevB.97.134508.

[ref134] Ginzburg V. Once again about high-temperature superconductivity. Contemporary Physics 1992, 33, 15–23. 10.1080/00107519208219137.

[ref135] Drozdov A.; Eremets M.; Troyan I.; Ksenofontov V.; Shylin S. I. Conventional superconductivity at 203 K at high pressures in the sulfur hydride system. Nature 2015, 525, 73–76. 10.1038/nature14964. 26280333

[ref136] Sun Y.; Lv J.; Xie Y.; Liu H.; Ma Y. Route to a superconducting phase above room temperature in electron-doped hydride compounds under high pressure. Physical review letters 2019, 123, 097001 10.1103/PhysRevLett.123.097001. 31524448

[ref137] Peng F.; Sun Y.; Pickard C. J.; Needs R. J.; Wu Q.; Ma Y. Hydrogen clathrate structures in rare earth hydrides at high pressures: possible route to room-temperature superconductivity. Physical review letters 2017, 119, 107001 10.1103/PhysRevLett.119.107001. 28949166

[ref138] Song P.; Hou Z.; Castro P. B. d.; Nakano K.; Hongo K.; Takano Y.; Maezono R. High-T c Superconducting Hydrides Formed by LaH24 and YH24 Cage Structures as Basic Blocks. Chem. Mater. 2021, 33, 9501–9507. 10.1021/acs.chemmater.1c02371.

[ref139] Nitz W. R. In Medizintechnik: Verfahren - Systeme - Informationsverarbeitung; Kramme R., Ed.; Springer, Berlin, Heidelberg, 2017.

[ref140] Zeng R.; Murashov V.; Beales T.; Liu H.; Dou S. High temperature superconducting magnetic levitation train. Appl. Supercond. 1997, 5, 201–204. 10.1016/S0964-1807(98)00026-X.

[ref141] Stekly Z.; Zar J. Stable superconducting coils. IEEE Trans. Nucl. Sci. 1965, 12, 367–372. 10.1109/TNS.1965.4323653.

[ref142] Smith P.; Lewin J. Pulsed superconducting magnets for proton synchotrons. Nuclear Instruments and Methods 1967, 52, 298–308. 10.1016/0029-554X(67)90235-2.

[ref143] Beenakker C. Annihilation of colliding Bogoliubov quasiparticles reveals their Majorana nature. Physical review letters 2014, 112, 070604 10.1103/PhysRevLett.112.070604. 24579584

[ref144] Leijnse M.; Flensberg K. Introduction to topological superconductivity and Majorana fermions. Semicond. Sci. Technol. 2012, 27, 124003 10.1088/0268-1242/27/12/124003.

[ref145] Qi X.; Hughes T.; Raghu S.; Zhang S. Time-Reversal-Invariant Topological Superconductors and Superfluids in Two and Three Dimensions. Phys. Rev. Lett. 2009, 102, 187001 10.1103/PhysRevLett.102.187001. 19518900

[ref146] Srednicki M. In Quantum Field Theory; Cambridge University Press, 2007.

[ref147] Björnson K. Topological band theory and Majorana fermions: With focus on self-consistent lattice models. Ph.D. Dissertation, Uppsala University, 2016.

[ref148] The Collected Works of Eugene Paul Wigner: Part A: The Scientific Papers; Wightman A. S., Ed.; Springer, Berlin, Heidelberg, 1993; p 213–226.

[ref149] Scaffidi T. In Weak-Coupling Theory of Topological Superconductivity: The Case of Strontium Ruthenate; Springer, 2017.

[ref150] Kohn W.; Luttinger J. M. New mechanism for superconductivity. Phys. Rev. Lett. 1965, 15, 524–526. 10.1103/PhysRevLett.15.524.

[ref151] Scalapino D.; Schrieffer J.; Wilkins J. Strong-coupling superconductivity. I. Phys. Rev. 1966, 148, 263–279. 10.1103/PhysRev.148.263.

[ref152] Landau L. D. On the theory of the Fermi liquid. Sov. Phys. JETP 1959, 8, 70.

[ref153] Neilson D. Landau Fermi liquid theory. Australian journal of physics 1996, 49, 79–102. 10.1071/PH960079.

[ref154] Eliashberg G. M. Interactions between electrons and lattice vibrations in a superconductor. Sov. Phys. JETP 1960, 11, 696–702.

[ref155] Naren H. R.; Thamizhavel A.; Nigam A. K.; Ramakrishnan S. Strongly correlated superconductivity in Rh 17 S 15. Physical review letters 2008, 100, 026404 10.1103/PhysRevLett.100.026404. 18232894

[ref156] Bauer E.; Rogl G.; Chen X.-Q.; Khan R. T.; Michor H.; Hilscher G.; Royanian E.; Kumagai K.; Li D. Z.; Li Y. Y.; Podloucky R.; Rogl P. Unconventional superconducting phase in the weakly correlated noncentrosymmetric Mo3Al2C compound. Phys. Rev. B 2010, 82, 064511 10.1103/PhysRevB.82.064511.

[ref157] Ruan B.-B.; Sun J.-N.; Chen Y.; Yang Q.-S.; Zhao K.; Zhou M.-H.; Gu Y.-D.; Ma M.-W.; Chen G.-F.; Shan L.; Ren Z.-A. Strong-coupling superconductivity with Tc ∼ 10.8 K induced by P doping in the topological semimetal Mo5Si3. Science China Materials 2022, 65, 3125–3133. 10.1007/s40843-022-2102-8.

[ref158] Fu L. Odd-parity topological superconductor with nematic order: Application to Cu_*x*_Bi_2_Se_3_. Phys. Rev. B 2014, 90, 100509 10.1103/PhysRevB.90.100509. 20868184

[ref159] Sasaki S.; Kriener M.; Segawa K.; Yada K.; Tanaka Y.; Sato M.; Ando Y. Topological superconductivity in cu x bi 2 se 3. Physical review letters 2011, 107, 217001 10.1103/PhysRevLett.107.217001. 22181913

[ref160] Yonezawa S. Bulk Topological Superconductors. ArXiv 2016, 1 10.22661/AAPPSBL.2016.26.3.03.

[ref161] Ono S.; Yanase Y.; Watanabe H. Symmetry indicators for topological superconductors. Physical Review Research 2019, 1, 013012 10.1103/PhysRevResearch.1.013012.

[ref162] He W.-Y.; Zhou B. T.; He J. J.; Yuan N. F.; Zhang T.; Law K. T. Magnetic field driven nodal topological superconductivity in monolayer transition metal dichalcogenides. Communications Physics 2018, 1, 40 10.1038/s42005-018-0041-4.

[ref163] Slager R.-J.; Juričić V.; Roy B. Dissolution of topological Fermi arcs in a dirty Weyl semimetal. Phys. Rev. B 2017, 96, 201401 10.1103/PhysRevB.96.201401.

[ref164] Shindou R.; Matsumoto R.; Murakami S.; Ohe J.-i. Topological chiral magnonic edge mode in a magnonic crystal. Phys. Rev. B 2013, 87, 174427 10.1103/PhysRevB.87.174427.

[ref165] Rüegg A.; Fiete G. A. Topological insulators from complex orbital order in transition-metal oxides heterostructures. Phys. Rev. B 2011, 84, 201103 10.1103/PhysRevB.84.201103.

[ref166] Pesin D.; MacDonald A. H. Spintronics and pseudospintronics in graphene and topological insulators. Nature materials 2012, 11, 409–416. 10.1038/nmat3305. 22522641

[ref167] Li Z.; Wei B. Topological materials and topologically engineered materials: properties, synthesis, and applications for energy conversion and storage. Journal of Materials Chemistry A 2021, 9, 1297–1313. 10.1039/D0TA11072H.

